# Exogenous sorbitol-chelated calcium mitigates toxicity of cadmium in peanut seedlings through physiological, biochemical, and transcriptomic regulation

**DOI:** 10.3389/fpls.2026.1741995

**Published:** 2026-03-23

**Authors:** Li Zhao, Yuzhao Cui, Yudong Chen, Fei Wang, Ruili Zheng, Mingxia Zhang, Shiman Li, Yafei Li, Cunzhen Geng, Dongyun Yan

**Affiliations:** 1 School of Environment and Geography, Qingdao University, Qingdao, China; 2 State Environmental Protection Key Laboratory of Soil Environmental Management and Pollution Control, Nanjing Institute of Environmental Sciences, Ministry of Ecology and Environment, Nanjing, China

**Keywords:** antioxidant defence, cadmium stress, peanut seedlings, sorbitol-chelated calcium, transcriptomic analysis

## Abstract

**Introduction:**

Soil cadmium (Cd) contamination is considered to be one of the adverse stresses to which plants are subject. Research has demonstrated that exogenous calcium plays a crucial role in plant stress resistance.

**Methods:**

Here, a controlled comparative experiment was conducted using peanut seedlings (*Arachis hypogaea* L.) exposed to Cd stress and supplied with either inorganic calcium or sorbitol-chelated calcium (SCC) at an equivalent calcium (Ca) concentration. This investigation was undertaken through integrated physiological, biochemical and transcriptomic analyses.

**Results:**

In the context of Cd stress, a marked inhibition in the growth parameters, photosynthetic activity, and root architecture of peanut seedlings was observed. This inhibition resulted in a significant accumulation of reactive oxygen species (ROS) within the plants. The application of exogenous calcium has been demonstrated to effectively alleviate Cd toxicity, with SCC exhibiting particularly notable efficacy in this regard. In comparison with Cd treatment, SCC significantly improved plant growth parameters and photosynthetic efficiency. Furthermore, SCC significantly enhanced superoxide dismutase (SOD) activity in tissues while concomitantly reducing malondialdehyde (MDA) and ROS levels, thereby mitigating membrane lipid oxidation. Concurrently, the analysis revealed that the SCC samples exhibited an upregulation of key genes, including AUX/IAA, GH3, SAUR, and JAZ. These genes have been implicated in promoting root growth and activating defence-related hormone pathways. Structural equation modelling further indicated that chlorophyll fluorescence exerted a significant positive influence on biomass accumulation, while excessive reactive oxygen species and osmotic regulators served as major inhibitory factors.

**Discussion:**

Consequently, SCC effectively mitigates Cd toxicity by stabilising photosynthetic systems, enhancing antioxidant defences, and regulating hormonal signalling, thereby promoting recovery of peanut seedling growth. The present study offers novel insights and a scientific basis for the efficient utilisation of Ca-containing fertilisers and the mitigation of heavy metal pollution in agricultural fields.

## 1 Introduction

Cadmium (Cd) is a highly toxic and non-biodegradable heavy metal that can be easily absorbed by crops and accumulate in them (Fan et al., 2024). Industrial emissions, mining activities, wastewater irrigation, and the long-term application of fertilizers that contain Cd all contribute to the accumulation of Cd in the soil (Zulfiqar et al., 2022). The buildup of Cd not only disturbs microbial communities, decreases enzyme activity, and damages soil fertility but also substantially increases the exposure risk of the plants to Cd (Zhang et al., 2015). Cd enters roots through both passive diffusion (apoplastic pathway) and active transport (symplastic pathway) (Yang et al., 2022). It is subsequently transported through the xylem to aboveground tissues, where it accumulates (Adil et al., 2020). These processes severely impair plant growth, development and metabolism, mainly by suppressing photosynthesis and respiration as well as perturbing nitrogen and carbohydrate metabolism (Haider et al., 2021; Wani et al., 2017; Chen G, et al., 2023). Moreover, Cd can accumulate in edible tissues and enter the human food chain, potentially causing irreversible damage to kidneys, bones, and other vital organs, representing a serious public health concern (Huang et al., 2024; Peana et al., 2023; Zeeshan et al., 2021). Therefore, exploring effective strategies to lower the toxicity of Cd and enhance the tolerance of crops to this heavy metal is highly important for safety of food and development of sustainable agriculture.

Calcium (Ca) is one of the essential macronutrients required by plants and plays an indispensable role in maintaining cellular structure and function. As an important component of the cell wall, Ca forms “calcium bridges” with pectin, which substantially elevates the strength and stability of the cell wall (Pei et al., 2018). At the membrane level, Ca helps to regulate the stability of the phospholipid bilayer, reduces the peroxidation of membrane lipids (Cramer et al., 1985), and maintains integrity and fluidity of the membrane (Narwal et al., 2024), thereby improving the plant’s ability to adapt to environmental stresses. In addition, Ca acts as a secondary messenger in intracellular signal transduction. It activates a series of stress-response signalling pathways that trigger antioxidant, defence, and repair mechanisms by regulating the activity of key enzymes, such as calcium-dependent protein kinases (CDPKs) (Khan et al., 2020). The protective role of Ca becomes particularly evident under Cd stress. On the one hand, Ca can reduce the uptake of Cd by the roots, suppress its movement within cells, and minimize its interference with cellular metabolism (Li et al., 2021). Alternatively, Ca strengthens the activities of antioxidant enzymes, such as superoxide dismutase (SOD), catalase (CAT), and peroxidase (POD), promotes the removal of excess reactive oxygen species (ROS), and alleviates oxidative damage to the cells (Wang et al., 2014; Huang et al., 2022). In addition, Ca helps maintain intracellular ion homeostasis, reducing Cd-induced ion toxicity (Korenkov et al., 2007; Weng et al., 2022). Moreover, Ca plays an irreplaceable role in stabilizing the structure and function of proteins and enzymes, which is essential for maintaining normal cellular metabolism. However, Ca in the soil is easily fixed and therefore difficult for crops to absorb effectively. This limits its efficiency in agricultural production.

In recent years, chelated Ca has attracted increasing attention in agricultural research because of its advantages in promoting nutrient absorption, improving yield and quality, and enhancing stress tolerance in crops. Studies have shown that the foliar application of sorbitol-chelated calcium (SCC) can significantly increase the photosynthetic capacity, yield, and quality of peanut (*Arachis hypogaea* L.), and promote the accumulation of Ca and magnesium in kernels (Li et al., 2022b). The most effective concentration of Ca was between 1.6 and 1.8 g/L. In potato (*Solanum tuberosum* L.), foliar spraying with Ca chelated with sugar alcohol not only improved the yield of tubers and marketable ratio but also enhanced the accumulation of nutrients, such as Ca, N, phosphorus, and potassium in the tubers (Li et al., 2020). The absorption and translocation efficiency of self-made chelated calcium fertilizer were higher than those of commercial products. Under NaCl stress, soaking rapeseed (*Brassica napus* L.) seeds with SCC effectively alleviated the inhibition of seed germination, and treatments with a 7:3 ratio of chelated to free Ca were more favourable at boosting the vigour of germination and radicle growth (Wei et al., 2022). Compared with the ionic forms, chelated mineral elements were more effective at enhancing stress resistance in plants. For example, foliar spraying with EDTA-chelated calcium was found to considerably reduce the accumulation of Cd in peanut kernels, and the maximum reduction was 40.3%. This effect may be related to the enhanced retention of Cd in the stems and leaves, the reduced translocation of Cd to the kernels, and changes in its subcellular distribution (Li et al., 2025). Further studies also demonstrated that nutrients chelated with sugar alcohols, such as chelated zinc and chelated iron, can activate antioxidant enzymes in rice (*Oryza sativa* L.) under Cd stress, which significantly enhanced the plant’s ability to eliminate ROS and alleviate the cell damage caused by oxidative stress (Li, 2023).

The studies described above have showed the potential of SCC for reducing the accumulation of Cd in crops, but its roles in alleviating the oxidative stress induced by Cd and regulating the Ca signalling pathways remain unclear. The physiological mechanisms by which SCC regulates the plant responses to Cd stress merit requires further study. In recent years, transcriptomic technology has become an important tool to study the plant responses to environmental stresses. Analysing the changes in profiles of gene expression enables a transcriptome study to help reveal the molecular regulatory networks involved in the adaptation of plants to stress conditions (Aslam et al., 2024). This approach has been widely applied in researches on low temperature, salinity (Jia et al., 2022), and drought stresses (Wei et al., 2024) and has provided valuable insights for the genetic improvement of crops and enhancement of stress tolerance. Under heavy metal stress, transcriptomic analyses have been used to explore the molecular mechanisms that underlie the plant responses. For example, studies on peanut under manganese stress have shown great differences in gene expression between the roots and leaves, particularly in antioxidant systems and metal ion homeostasis (Liu et al., 2023). Similarly, comparative analyses of peanut cultivars with contrasting levels of Cd accumulation have identified key genes related to the transport and sequestration of Cd, which indicated that low-Cd cultivars can more effectively restrict Cd within the roots and leaves (Yu et al., 2018). Moreover, studies have revealed that treatments, such as combined magnetite/zinc oxide (Fe_3_O_4_/ZnO) nanoparticles, can mitigate Cd toxicity by strengthening the expression of genes related to antioxidants, reducing the accumulation of Cd, and improving plant tolerance (Wang et al., 2025). These findings suggest that transcriptomic analyses provide important theoretical support to understand plant stress resistance and has become a vital method to study the mechanisms of tolerance to heavy metals.

Therefore, this study systematically investigated the effects of chelated Ca and free Ca on improving the tolerance of peanut seedlings to Cd stress by combining physiological and biochemical analyses with transcriptomic approaches. The aim was to clarify the mechanisms by which chelated Ca alleviates Cd toxicity, provide theoretical support for the development of efficient Ca fertilizers, enhance the tolerance of crops to Cd, and offer a scientific basis for the remediation of heavy metal pollution in agricultural soils.

## 2 Materials and methods

### 2.1 Preparation of SCC

Sorbitol was first dissolved in deionized water, and Ca nitrate was added at a sorbitol-to-calcium nitrate molar ratio of 2:1. The mixture was then stirred magnetically for 35 min at 65 °C to facilitate the chelation reaction. After cooling, a clear and transparent chelated stock solution was obtained. The chelation rate was determined as previously described (Cui et al., 2022), and the results from three independent measurements were all close to 100%. This indicated that Ca primarily existed in the chelated form.

### 2.2 Growth conditions of peanut seedlings and preparation of polluted substrate

The peanut cultivar ‘Huayu 22’ was used in this study. The seeds were surface-sterilized and pre-cultured in moist quartz sand (16–20 mesh) for 3 days. After the seedlings had developed two true leaves, they were transplanted into seedling trays containing Hoagland nutrient solution (pH 6.5). The substrate that contained Cd was prepared by evenly spraying quartz sand with a solution of cadmium chloride (CdCl_2_) to prepare a concentration of approximately 2.5 mg/kg Cd. The treated sand was then sealed and aged for 15 days before use.

### 2.3 Experimental design

Seedlings that reached the three-leaf stage were transplanted into pots (9.3 cm in diameter and 12 cm high) that each contained 450 g of sterilized quartz sand. Every 2 days, 50 mL of nutrient solution corresponding to each treatment was added to the pots. The concentration of Ca was established from preliminary research, and 5 mM was found to be optimal (**Supplementary Table 1**). Four treatments were established, and there were three replicates of each treatment:

1. CK — 1/2 Hoagland nutrient solution;
2. Cd — 1/2 Hoagland nutrient solution with Cd;
3. Cd_CN — 1/2 Hoagland nutrient solution with Cd and 5 mM calcium nitrate;
4. Cd_SCC — 1/2 Hoagland nutrient solution with Cd and 5 mM sorbitol-chelated calcium.

In total, 12 pots were arranged randomly and repositioned daily to maintain consistent growth conditions. The cultivation environment was set as follows: light intensity of 800 µmol·m^-^²·s^-^¹ with a 14 h light/10 h dark photoperiod, day/night temperatures of 26/20 °C, and relative humidity of 60 ± 5%. The seedlings were harvested for subsequent physiological, biochemical, and transcriptomic analyses after 15 days of treatment.

### 2.4 Measurement of the growth parameters and root morphology

The plant height and primary root lengths were measured using a ruler (1 mm precision), and the stem diameter at the base was measured with a Vernier calliper. The plants were carefully washed with deionized water and separated into roots, stems, and leaves using stainless steel scissors. A portion of the samples were dried in a forced-air oven at 105 °C for 30 min to deactivate the enzymes and then dried to a constant weight at 75 °C to determine the dry mass of each organ. The remaining samples were frozen in liquid nitrogen and stored at −80 °C for subsequent physicochemical and transcriptomic analyses. The roots were rinsed 3–5 times with deionized water and scanned using a root scanner (V700 Photo, Epson, Japan) to analyse the root morphology. The images obtained were analysed with WinRHIZO™ 2003b software (Regent Instruments, Quebec, Canada) to determine the total root length, root surface area, root volume, average diameter, and number of root tips.

### 2.5 Measurement of the photosynthetic parameters and content of chlorophyll

A portable photosynthesis system (Li-6400, Li-Cor Inc., USA) was used to measure the photosynthetic parameters on clear and windless mornings before the crop was harvested. Measurements were taken from the third leaf from the top of the main stem to determine the net photosynthetic rate (Pn, μmol·m^-^²·s^-^¹), transpiration rate (Tr, μmol·m^-^²·s^-^¹), intercellular CO_2_ concentration (Ci, μL·L^-^¹), and stomatal conductance (Gs, μmol·m^-^²·s^-^¹). Three plants were measured for each treatment.

The chlorophyll fluorescence parameters were determined using a chlorophyll fluorescence imaging system (Imaging-PAM, WALZ Inc., Germany). The initial fluorescence (F_0_), maximum quantum yield of Photosystem II (Fv/Fm), effective quantum yield of PSII (ΦPSII), photochemical quenching coefficient (qP), non-photochemical quenching coefficient (qN), and electron transport rate (ETR) were recorded.

The pigments were analysed by extracting the chlorophyll with 80% acetone, and the concentrations of chlorophyll a, chlorophyll b, total chlorophyll, Chl a/b ratio, and carotenoids were calculated as previously described (Song et al., 2020).

### 2.6 Determination of the contents of ROS and malondialdehyde

The contents of superoxide anion (O_2_^-^) and hydrogen peroxide (H_2_O_2_) were determined using the hydroxylamine hydrochloride oxidation method and the titanium sulfate colorimetric method, respectively. All reagents were provided by Solarbio Science & Technology Co., Ltd. (Beijing, China). The absorbance for O_2_^-^ was measured at 530 nm, and that for H_2_O_2_ was measured at 415 nm using a UV–Vis spectrophotometer (UV-2400, Shimadzu Corporation, Japan). The final concentrations of O_2_^-^ and H_2_O_2_ in the samples were calculated as previously described.

The content of malondialdehyde was determined by homogenizing 0.5 g of fresh tissue in 5 mL of 5% thiobarbituric acid (TBA) using a pre-chilled mortar and pestle (Papastergiadis et al., 2012). The homogenate was centrifuged at 4500 g for 15 min at 4 °C. A volume of 2 mL of the supernatant was then mixed with 2 mL of 0.67% TBA in a glass tube, boiled in a water bath for 30 min, cooled, and centrifuged again at 4500 g for 15 min.

### 2.7 Determination of the contents of antioxidants and activities of antioxidant enzymes

The content of soluble sugar was determined using the anthrone–sulfuric acid colorimetric method (Li et al., 2000); that of the soluble protein was measured using the Coomassie brilliant blue G-250 method (Ashraf and Foolad, 2007), and that of proline was obtained using the sulfosalicylic acid method (Bates et al., 1973). The activities of antioxidant enzymes, including SOD, POD, and CAT, were measured using commercial assay kits (Solarbio Science & Technology Co., Ltd., Beijing, China) according to the manufacturer’s instructions.

### 2.8 RNA extraction, sequencing, and *de novo* assembly

Total RNA was extracted from peanut seedling roots using TRIzol^®^ reagent (Invitrogen, Carlsbad, CA, USA). The RNA concentration, purity, and integrity were assessed using a NanoDrop 2000 spectrophotometer (Thermo Fisher Scientific, USA), agarose gel electrophoresis, and an Agilent 5300 Bioanalyzer (Agilent Technologies, USA) (Liu and Zhu, 2020). Samples with an RNA Quality Number (RQN) > 6.5, OD260/280 between 1.8 and 2.2, OD260/230 ≥ 2.0, 28S:18S ratio ≥ 1.0, and a total RNA amount ≥ 1 μg were selected for library construction.

High-quality RNA samples were sent to Shanghai Majorbio Bio-pharm Technology Co., Ltd. (Shanghai, China) for RNA sequencing (RNA-seq), which was performed on an Illumina NovaSeq X Plus using paired-end 150 bp reads. High-quality clean reads were obtained by conducting data quality control using fastp (https://github.com/OpenGene/fastp). This step included the removal of adapter sequences, low-quality bases (Q < 20), and reads that contained more than 10% ambiguous bases and were < 20 bp.

The clean RNA-seq reads were aligned to the *Arachis hypogaea* L. reference genome (http://peanutgr.fafu.edu.cn/Download.php.) using HISAT2 (http://ccb.jhu.edu/software/hisat2/index.shtml), and transcripts were assembled with StringTie (https://ccb.jhu.edu/software/stringtie/).

### 2.9 Annotation of gene function and analysis of the differentially expressed gene

The unigene sequences were compared against several public databases using the BLASTX program, including the NCBI non-redundant protein database (NR), the Clusters of Orthologous Groups (COG), the Protein Families database (Pfam), the Kyoto Encyclopedia of Genes and Genomes (KEGG), Gene Ontology (GO), and the Swiss-Prot protein database. The corresponding gene functions, classifications, and protein annotations were then obtained from these alignments (Chen et al., 2021).

The differentially expressed genes (DEGs) between the different treatments were identified by quantifying the abundance of transcripts using RSEM (http://deweylab.github.io/RSEM/), and the levels of expression were expressed as Fragments Per Kilobase of transcript per Million mapped reads (FPKM) or Transcripts Per Million (TPM) values. DESeq2 (http://bioconductor.org/packages/stats/bioc/DESeq2/) was used to differentially analyse the expression based on the alignment results. Genes with |log_2_(Fold Change)| ≥ 2 and FDR < 0.05 were considered significantly and differentially expressed. Venn diagrams were generated to illustrate the overlap and unique patterns of expression of DEGs among the treatments. GO (http://www.geneontology.org/) and KEGG (http://www.genome.jp/kegg/) enrichment analyses were performed to identify the major biological processes and pathways associated with these DEGs.

### 2.10 Construction of a structural equation model

The physiological parameters biomass, photosynthesis, root architecture, ROS, chlorophyll fluorescence, and osmotic regulation were categorized into six groups through a principal component analysis (PCA). The transcriptomic indicators were derived from selected gene sets (hormone, photo, and antiox). The TPM expression values of these genes across the 12 samples were standardized using z-score transformation, and the mean standardized values (MEAN-Z) were calculated and integrated as transcriptomic indicators.

Based on the logical framework of “signal regulation → root and photosynthetic effects → osmotic regulation → antioxidant system → biomass accumulation,” a structural equation model (SEM) was constructed. The SEM analysis was implemented using Stata MP 18.0 software (StataCorp, College Station, TX, USA), and the parameters were estimated by the maximum likelihood (ML) method. The model fit was evaluated using a chi-square (χ²) test, comparative fit index (CFI), Tucker–Lewis index (TLI), and root mean square error of approximation (RMSEA).

### 2.11 Statistical analysis

The data were recorded using Microsoft Excel 2021 (Microsoft, Redmond, WA, USA) and assessed statistically using SPSS 26.0 (IBM, Inc., Armonk, NY, USA). All the data were tested for normality and homogeneity of variance before analysis. A one-way analysis of variance (ANOVA) was performed, and a Duncan’s multiple range test was used to compare means at a significance level of *p*<0.05. The transcriptomic data were visualized using an online platform (https://cloud.majorbio.com/page/flow/index.html), while the other figures were generated using Origin 2021b (OriginLab, Northampton, MA, USA).

## 3 Results

### 3.1 Growth parameters of the peanut seedlings

**Figure 1:**
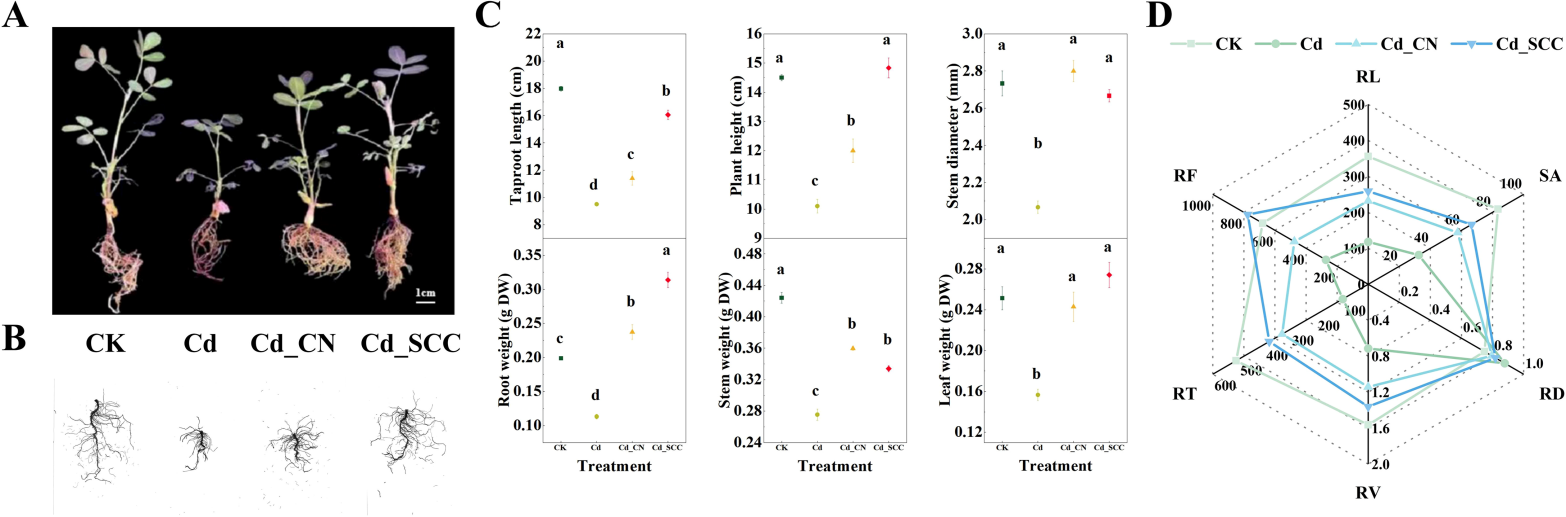
Effects of different calcium treatments on the morphology and physiological characteristics of peanut seedlings under Cd stress. **(A)** Growth performance; **(B)** root morphology; **(C)** growth parameters, including primary root length, plant height, stem diameter, and dry weights of roots, stems, and leaves; **(D)** root structural traits, including total root length (RL), root surface area (SA), average root diameter (RD), total root volume (RV), number of root tips (RT), and root forks (RF). Different letters indicate significant differences (*p*<0.05).

The treatment with Cd significantly inhibited the growth of peanut seedlings, which resulted in shorter plants and a reduction in root length (**Figures 1A, B**). The application of exogenous Ca, particularly SCC, effectively alleviated the inhibition of growth induced by Cd and promoted the overall development of plants and roots. A quantitative analysis further confirmed these visual observations (**Figures 1C, D**). Compared with the Cd treatment, the plant height and primary root length in the Cd_SCC group increased by 46.86% and 69.12%, respectively, while those in the Cd_CN group increased by 18.81% and 20.00%, respectively. In addition, treatment with exogenous Ca restored the stem diameter under Cd stress to a level comparable to that of the control (CK), and there was no distinct difference between the SCC and CN treatments. Both Ca treatments also promoted the accumulation of dry matter, and the root, stem, and leaf dry weights in the Cd_SCC treatment were 177.45%, 21.23%, and 75.03% higher than those in the Cd treatment, respectively.

In terms of root morphology (**Figure 1D**), Cd stress markedly inhibited the root growth and reduced the total root length, surface area, and volume by 66.81%, 61.05%, and 54.30% compared with CK, respectively, while the average root diameter increased by 17.26%. Exogenous Ca mitigated the adverse effects of Cd since the total root length in Cd_SCC and Cd_CN treatments rised by 119.43% and 96.40% compared with Cd alone, respectively. There were no significant differences between the two. Both the SCC and CN treatments substantially increased the root surface area and root volume (*p*<0.05), although SCC was slightly more effective. Moreover, both treatments reduced the average root diameter to a level similar to that of the CK.

### 3.2 Photosynthetic parameters and chlorophyll content

**Figure 2:**
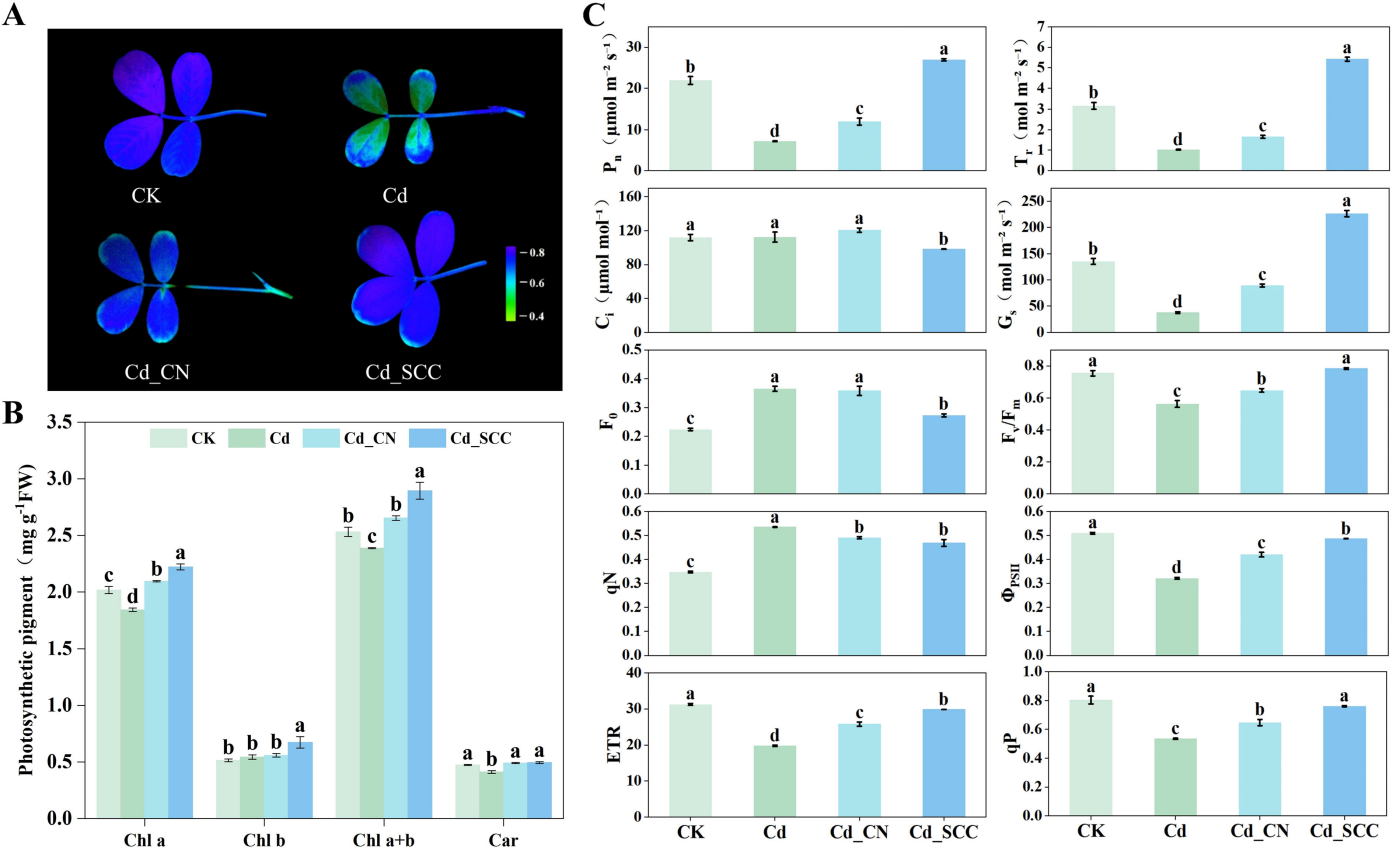
Effects of different treatments on the photosynthetic pigment content and chlorophyll fluorescence parameters of peanut leaves under Cd stress. **(A)** Chlorophyll fluorescence imaging; **(B)** photosynthetic pigment content of leaves; **(C)** photosynthetic parameters, including net photosynthetic rate, transpiration rate, intercellular CO_2_ concentration, stomatal conductance, initial fluorescence, maximum quantum efficiency of PSII, non-photochemical quenching, effective quantum yield of PSII, electron transport rate, and photochemical quenching. Different letters indicate significant differences (*p*<0.05).

Significant differences were observed in the chlorophyll fluorescence imaging of peanut leaves under the different treatments (**Figure 2A**). The control leaves appeared blue, which indicated a higher Fv/Fm value. Most of the leaves turned green under Cd stress, and only the edges remained blue. The Fv/Fm in the leaf veins was noticeably reduced. Only a small portion of the leaf margins appeared green in the Cd_SCC treatment, while the rest of the leaf recovered to a state similar to the control. In contrast, the leaves under the Cd_CN treatment had green edges and a mixture of blue and green in the inner regions.

Compared with the control, the Cd treatment dramatically decreased the contents of chlorophyll a, total chlorophyll, and carotenoids by 8.64%, 5.73%, and 13.41%, respectively, while the content of chlorophyll b grew by 5.64%. The application of exogenous Ca significantly enhanced the contents of chlorophyll a, chlorophyll b, total chlorophyll, and carotenoids. The content of chlorophyll a increased by 20.56% and 13.71% in the Cd_SCC and Cd_CN treatments, respectively, compared to the Cd treatment; chlorophyll b increased by 23.82% and 2.71%, respectively; and the total chlorophyll increased by 21.27% and 11.17%, respectively. The increasement in chlorophyll b was the most pronounced difference between the two Ca treatments. Both the Ca treatments also increased the contents of carotenoids, although the difference was not significant. Overall, SCC was more effective than calcium nitrate (**Figure 2B**).

Cd stress remarkably inhibited the net Pn of the peanut leaves and reduced it by 67.33% compared with the control. In contrast, the addition of SCC and CN significantly increased the Pn by 276.10% and 66.74%, respectively. The Cd treatment decreased the Tr, while exogenous Ca greatly improved it. Cd_SCC and Cd_CN increased the Tr by 430.94% and 61.39%, respectively, compared with the Cd treatment. Cd stress raised the intercellular CO_2_ concentration, whereas Cd_SCC significantly reduced the Ci by 12.68%. The Cd treatment also reduced the stomatal conductance by 72.10%, which was alleviated by exogenous Ca. Cd_SCC and Cd_CN increased the Gs by 500.54% and 135.86%, respectively. Overall, SCC more effectively improved the photosynthetic performance of the peanut leaves under Cd stress than calcium nitrate (**Figure 2C**).

The addition of exogenous Ca substantially affected the chlorophyll fluorescence parameters of the peanut leaves under Cd stress (**Figure 2C**). Compared with the control, the Cd treatment markedly increased the initial fluorescence. The F_0_ value decreased after the application of SCC and CN, and Cd_SCC was reduced by 25.23% compared with the Cd treatment, while the difference between Cd_CN and Cd was not significant. Cd stress reduced the maximum photochemical efficiency by 25.46%. In contrast, Cd_SCC and Cd_CN considerably increased the Fv/Fm by 39.50% and 15.12%, respectively, compared with Cd. Under the Cd treatment, the effective quantum yield of PSII decreased significantly, whereas both the SCC and CN treatments markedly improved ΦPSII. SCC was more effective at increasing it as shown by a 51.62% enhancement. Cd stress also reduced the photochemical quenching coefficient and ETR by 33.61% and 36.85%, respectively, compared to the CK. The application of SCC and CN significantly increased both parameters. The qP increased by 42.51% and 21.26% compared with the Cd treatment, and the ETR increased by 51.27% and 30.57% under Cd_SCC and Cd_CN, respectively. Cd stress dramatically increased the non-photochemical quenching coefficient, while the SCC and CN treatments decreased the qN, although there was no significant difference between the two Ca treatments.

### 3.3 Antioxidant system

#### 3.3.1 Effects on the accumulation of ROS

**Figure 3:**
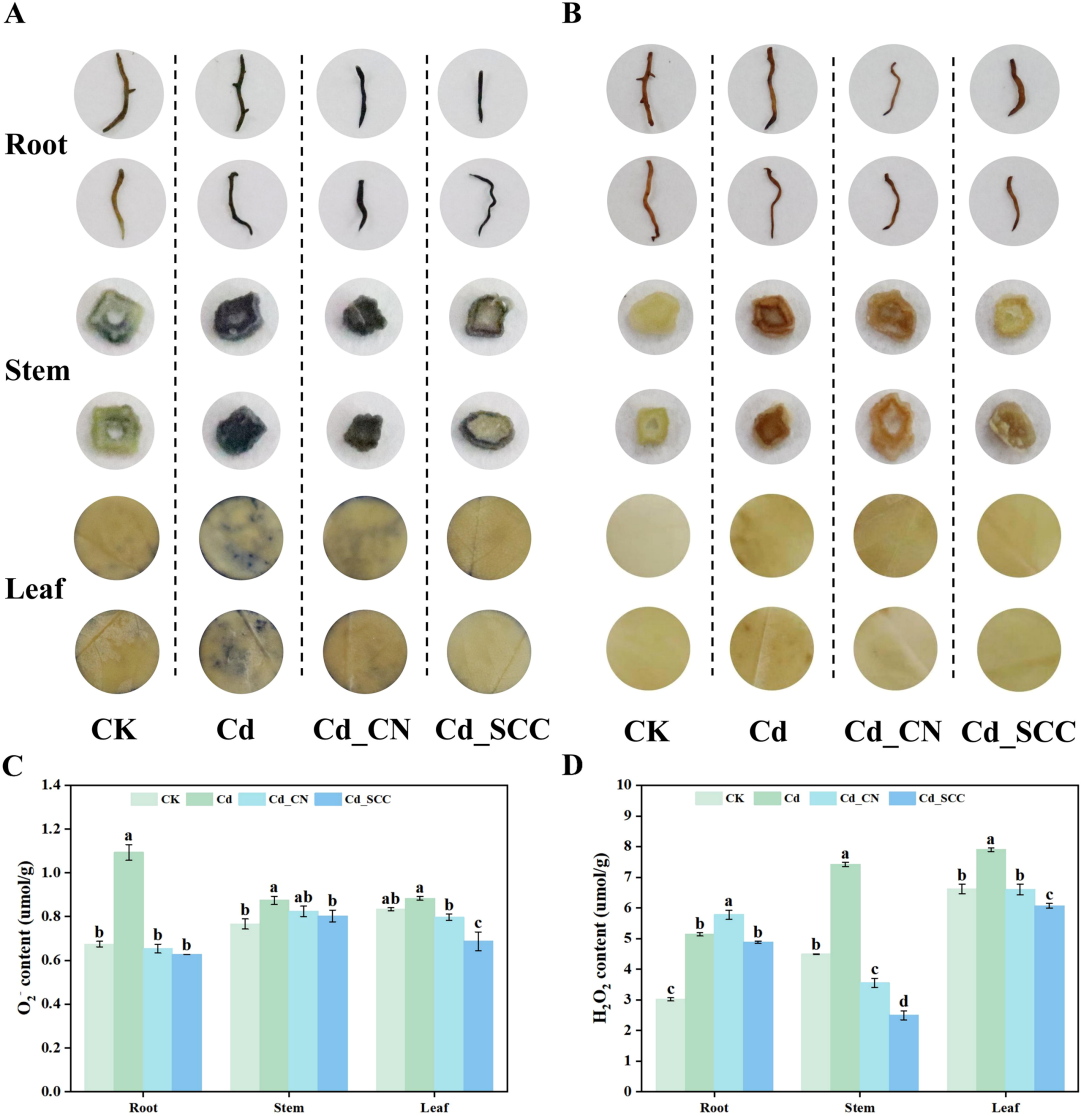
Effects of different treatments on O_2_^-^ and H_2_O_2_ accumulation in peanut seedlings under Cd stress. **(A)** Tissue staining of O_2_^-^; **(B)** tissue staining of H_2_O_2_; **(C)** O_2_^-^ content; **(D)** H_2_O_2_ content. Different letters indicate significant differences (*p*<0.05).

The effects of exogenous Ca on the accumulation of O_2_^-^ and H_2_O_2_ in peanut seedlings under Cd stress are shown in **Figures 3A, B**. After the Cd treatment, the roots, stems, and leaves stained the most, which indicated severe oxidative stress caused by the excessive accumulation of ROS. The addition of exogenous Ca reduced the intensity of staining, and the effect was more pronounced in the Cd_SCC treatment.

The quantitative analysis (**Figures 3C, D**) showed results consistent with the staining observations. The Cd treatment increased the content of O_2_^-^ in the roots, stems, and leaves by 62.04%, 14.10%, and 6.00%, respectively, and the most severe oxidative damage occurred in the roots. Exogenous Ca significantly reduced the accumulation of O_2_^-^. Compared with the Cd treatment, those of Cd_SCC and Cd_CN decreased the levels of O_2_^-^ in the roots by 42.62% and 40.21%, respectively, the levels in the stem by 8.12% and 5.72%, and the levels in the leaves by 22.26% and 9.80%, respectively. Similarly, Cd stress increased the content of H_2_O_2_ in the roots, stems, and leaves by 70.35%, 65.26%, and 19.41%, respectively. In contrast, the Cd_SCC treatment reduced the levels of H_2_O_2_ by 5.06%, 66.44%, and 23.17% in these organs, while the Cd_CN treatment reduced them by 52.07% and 16.46% in the stems and leaves but caused a 12.48% increase in the roots. Overall, the treatment with exogenous Ca alleviated the accumulation of ROS, and SCC was more effective at mitigation than calcium nitrate.

#### 3.3.2 Effects on the osmotic regulatory compounds and content of malondialdehyde

**Figure 4:**
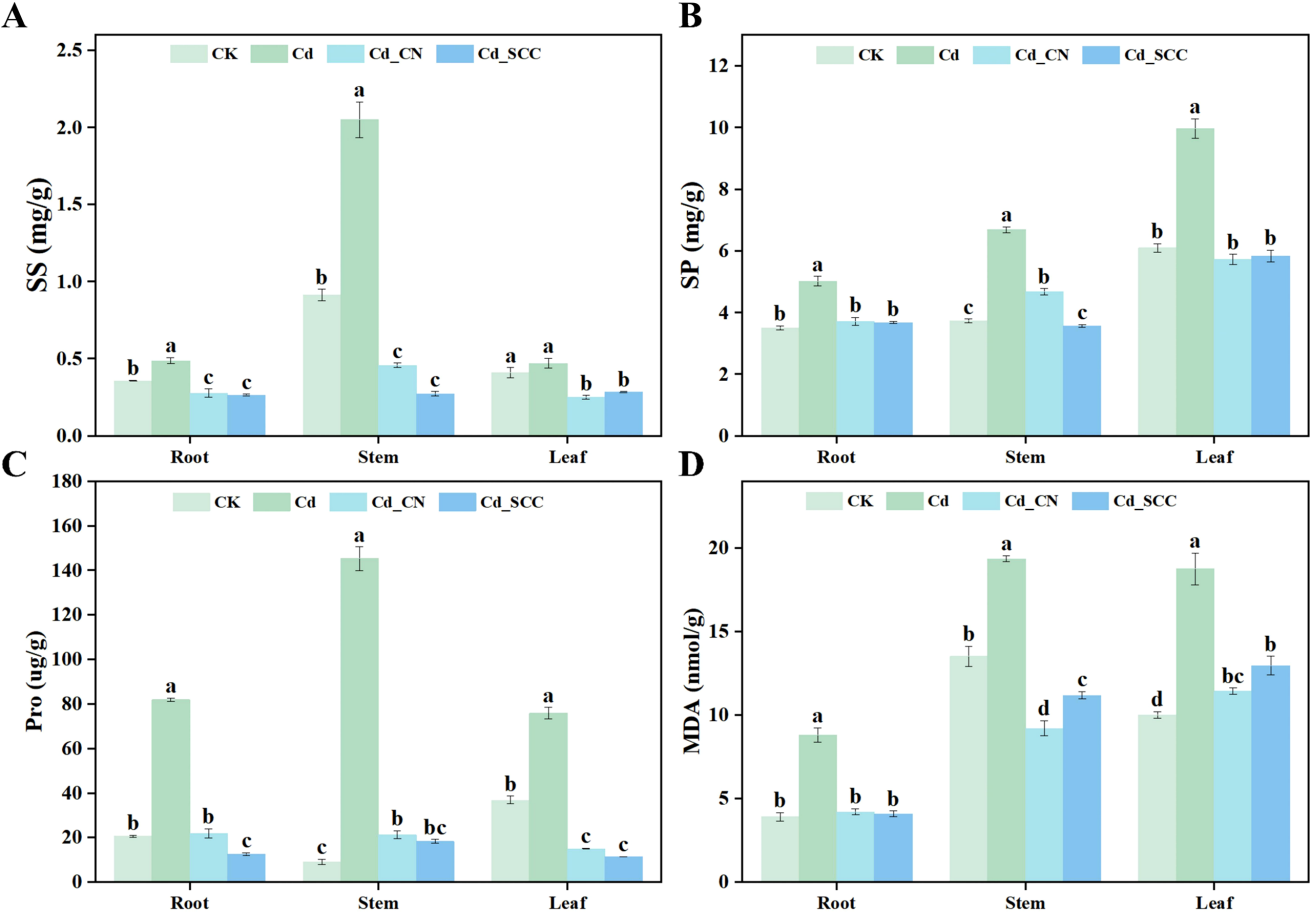
Effects of different treatments on the contents of osmotic regulatory substances in peanut seedlings under Cd stress. **(A)** SS, Soluble sugar; **(B)** SP: Soluble protein; **(C)** Pro: Proline; **(D)** MDA: Malondialdehyde. Different letters indicate significant differences (*p*<0.05).

Different treatments had significant effects on the osmotic regulatory compounds and content of MDA in peanut seedlings under Cd stress (**Figure 4**). The Cd stress markedly increased the content of soluble sugar (**Figure 4A**), whereas the application of exogenous Ca reduced it to a large extent. Compared with the Cd treatment, the Cd_SCC treatment decreased the content of soluble sugar in the roots, stems, and leaves by 45.90%, 86.67%, and 39.72%, respectively, while the Cd_CN treatment reduced it by 43.15%, 77.72%, and 46.81%, respectively. There was no significant difference between the two sources of Ca.

The variations in the contents of soluble protein and proline showed trends similar to those of soluble sugar. The contents of soluble protein and proline increased heavily in the roots, stems, and leaves after Cd exposure compared with the control. The contents of soluble protein increased by 43.33%, 79.52%, and 63.48%, respectively, while that of proline increased by 298.67%, 1511.14%, and 105.90%, respectively. The application of exogenous Ca significantly reduced the accumulation of soluble protein and proline induced by Cd, which restored them to levels comparable to the control. In the stems, the content of soluble protein differed greatly between the Cd_SCC and Cd_CN treatments. The content of proline in the roots under Cd_SCC was 42.44% lower than that under Cd_CN.

Cd stress also significantly increased the content of MDA in the roots, stems, and leaves (**Figure 4D**). Compared with the control, the content of MDA under the Cd treatment increased by 125.34%, 43.31%, and 87.28%, respectively, which indicated severe lipid peroxidation caused by Cd toxicity. Treatment with exogenous Ca effectively alleviated the accumulation of MDA induced by Cd. The levels of MDA in the roots, stems, and leaves treated with Cd_SCC decreased by 53.58%, 42.26%, and 30.93%, respectively, while the Cd_CN treatment reduced them by 52.37%, 52.45%, and 39.09%, respectively. There was a big difference between the two sources of Ca in the stems but not in the roots or leaves. Across the tissues, the content of MDA in the stems and leaves was generally higher than that in the roots. These results suggest that the treatment with exogenous Ca effectively mitigated the damage from the peroxidation of lipids induced by Cd in the cell membranes of peanut seedlings.

#### 3.3.3 Effects on the activities of antioxidant enzymes

**Figure 5:**
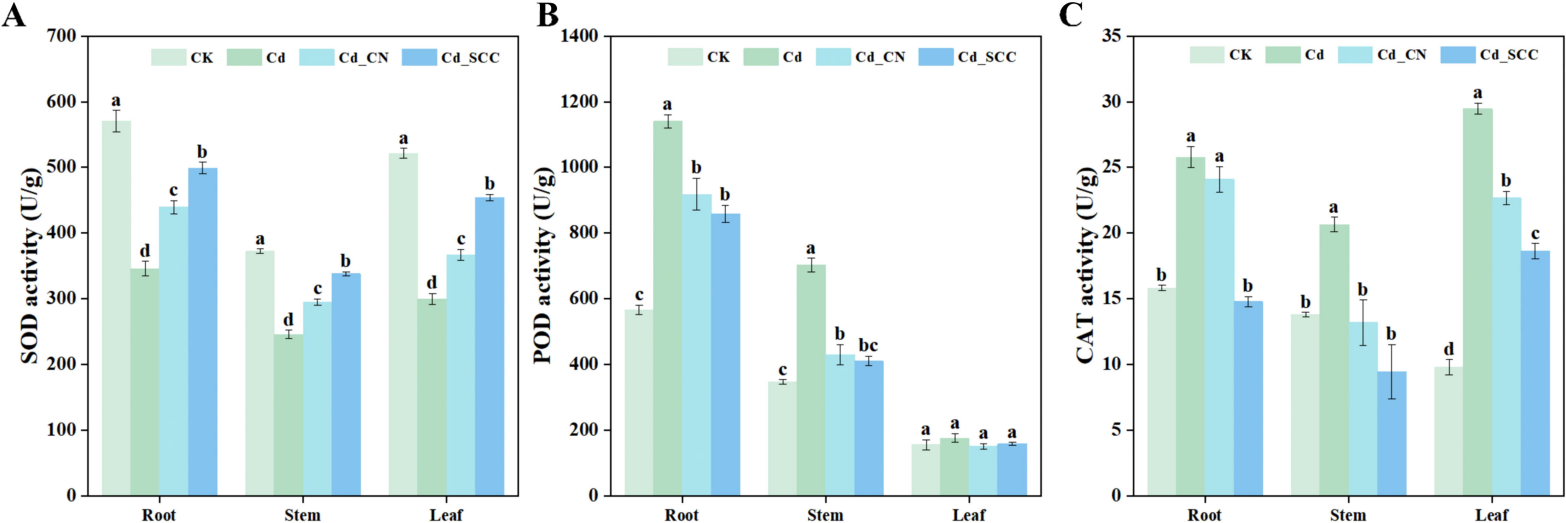
Effects of different treatments on enzyme activities in peanut seedlings under Cd stress. **(A)** SOD; **(B)** POD; **(C)** CAT. Different letters indicate significant differences (*p*<0.05).

Exogenous Ca significantly affected the antioxidant enzyme activities of peanut seedlings under Cd stress (**Figure 5**). The Cd treatment markedly inhibited the activity of SOD, which decreased by 39.33%, 34.02%, and 42.58% in the roots, stems, and leaves, respectively. The application of exogenous Ca significantly restored the activity of SOD, and the strongest effect was observed in the chelated Ca treatment (Cd_SCC). The activity of SOD in this treatment increased by 44.32%, 37.58%, and 51.78% in the roots, stems, and leaves, respectively. Cd_CN also improved the activity of SOD, but to a lesser extent. The values raised by 27.07%, 20.22%, and 22.72%, respectively.

Cd stress strongly enhanced the activity of POD and increased it by 101.27%, 102.92%, and 13.41% in the roots, stems, and leaves, respectively. Treatment with exogenous Ca reduced the activity of POD, while Cd_SCC decreased the activity of POD in the roots and stems by 24.69% and 41.63%, respectively, while no significant change was observed in the leaves. The Cd treatment also increased the activity of CAT by 62.81%, 49.84%, and 201.12% in the roots, stems, and leaves, respectively. The activity of CAT in the roots and stems deactivated to levels close to the CK after the addition of Ca. Cd_SCC reduced the activity of CAT in the roots and stems by 42.68% and 54.16%, respectively, compared with Cd.

There was no big difference in the activities of POD and CAT in the stems between the two Ca treatments, while all the other parameters differed significantly. Although Cd_CN also reduced the activity of CAT in the stems and leaves by 36.17% and 23.09%, respectively, Cd_SCC was more effective at alleviating it than Cd_CN.

### 3.4 Transcriptomic sequencing of the peanut roots

#### 3.4.1 RNA sequencing quality and sample consistency

A total of 12 transcriptome libraries were obtained in this study. The proportion of clean reads to raw reads ranged from 93.67% to 97.43%, with an average of 96.03%. The Q30 values were between 91.70% and 92.88% and averaged 92.44%. The average sequencing error rate was 0.027%, and the mean GC content was 45.66%. The overall mapping rate of reads to the reference genome ranged from 92.70% to 94.99%, with an average of 93.89%. This included an average unique mapping rate of 81.47% and a multiple mapping rate of 12.42%. These results revealed that there were both high-quality sequencing and alignment. Thus, they met the requirements for subsequent differential expression and functional enrichment analyses (**Supplementary Table 2**).

**Figure 6:**
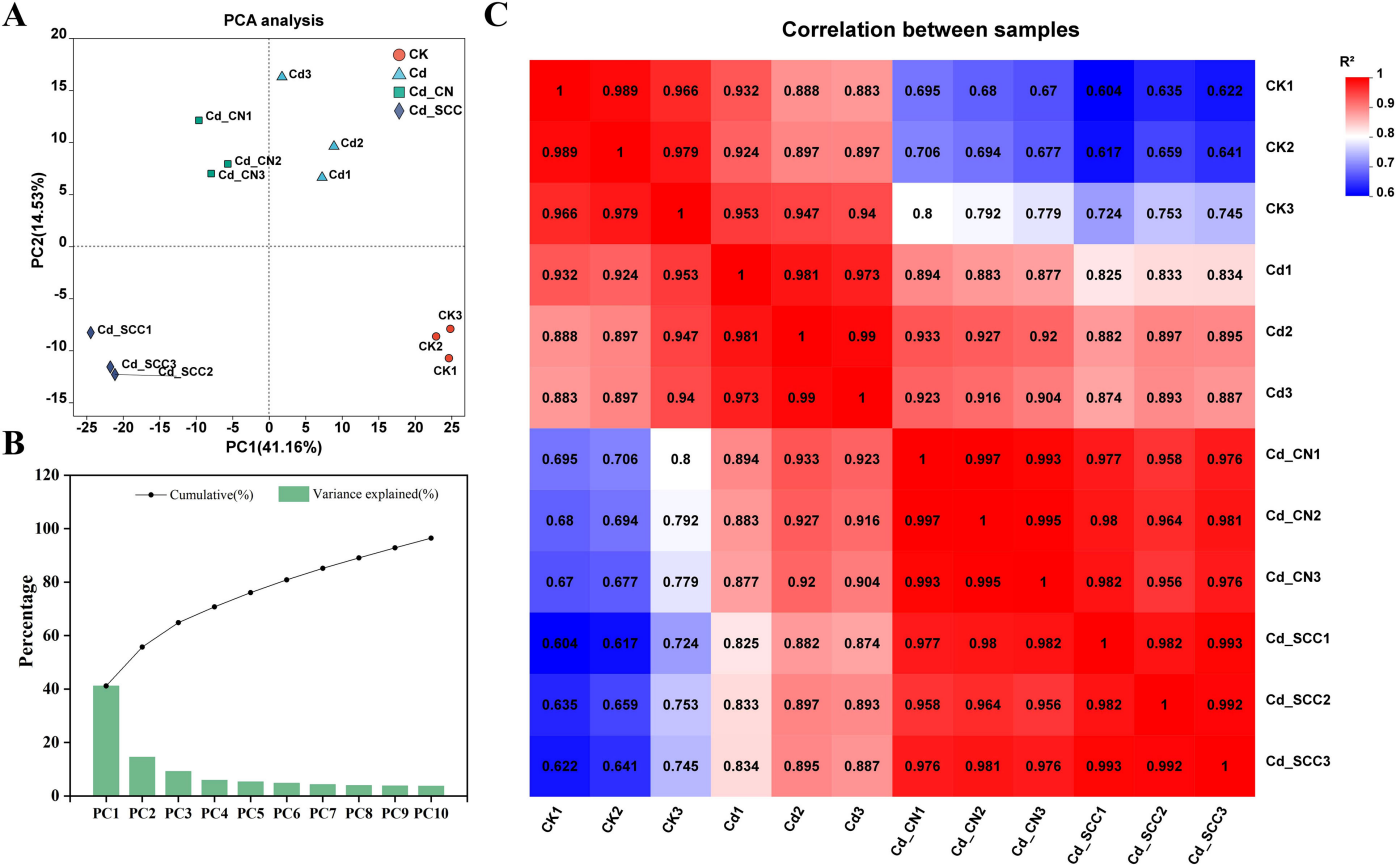
Consistency and grouping analysis of transcriptome samples from peanut roots. **(A)** PCA of overall gene expression levels; **(B)** contribution rates of principal components; **(C)** correlation heatmap of overall gene expression among samples.

A principal component analysis (PCA) was performed on the levels of gene expression in the peanut roots to further verify the consistency among the sequencing samples and the differences between treatments. The results showed clear separation among the treatments along the first two principal components, while the replicates within each treatment were tightly clustered (**Figure 6A**). The variance explained by the principal components (**Figure 6B**) revealed that PC1 and PC2 together accounted for 55.7% of the total variation. This suggests that the PCA effectively captured the differences in expression among the treatments. The correlation analysis among the samples (**Figure 6C**) showed that Pearson’s correlation coefficients (R²) between biological replicates was all > 96%. This further confirmed that the samples were consistent, and the transcriptome data were highly reliable. Thus, this provided a solid basis for subsequent analyses.

#### 3.4.2 Analysis of the DEGs

**Figure 7:**
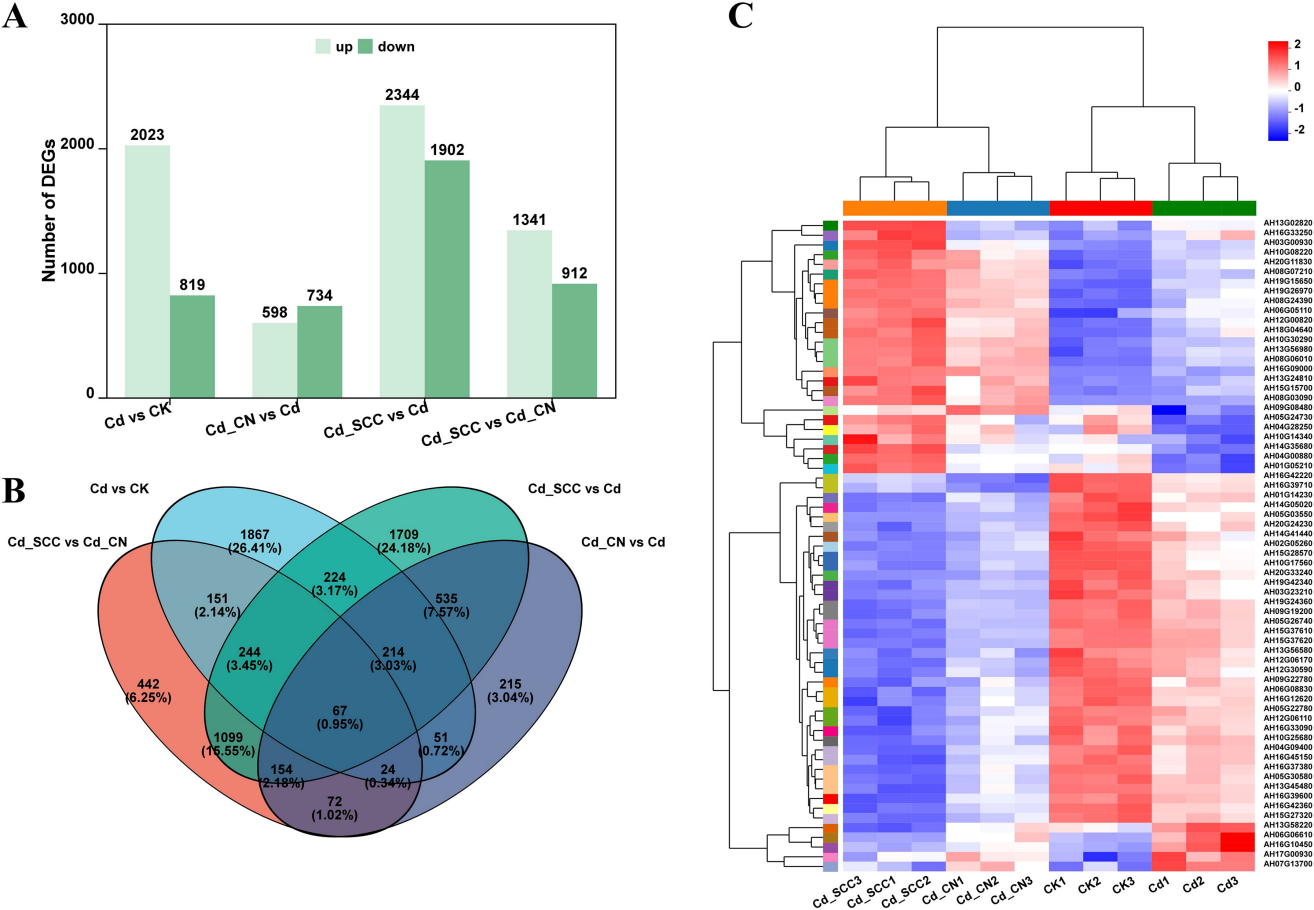
Statistical analysis of DEGs in peanut roots. **(A)** Number of differentially expressed genes; **(B)** Venn diagram of DEGs; **(C)** clustering heatmap of DEGs.

The DEGs in peanut roots under different treatments were identified using the criteria |log_2_(Fold Change)| ≥ 2 and FDR < 0.05 (**Figure 7A**). Compared with CK, Cd treatment resulted in 2,842 DEGs, including 2,023 upregulated and 819 downregulated genes. Compared with Cd treatment, 4,246 DEGs were identified in Cd_SCC (2,344 upregulated and 1,902 downregulated) and 1,332 DEGs in Cd_CN (598 upregulated and 734 downregulated). Between Cd_SCC and Cd_CN treatments, 2,253 DEGs were identified (1,341 upregulated and 912 downregulated). Overall, the number of DEGs induced by the chelated Ca was much higher than that induced by inorganic Ca, which suggests that SCC exerts broader transcriptional regulation.

The Venn diagram analysis (**Figure 7B**) revealed that each comparison contained both shared and unique DEGs, and 67 genes were differentially expressed across all the comparisons. A hierarchical clustering analysis (**Figure 7C**) showed that the biological replicates within each treatment were highly consistent, while the patterns of expression varied markedly among the treatments. Notably, many genes exhibited opposite trends in expression between the Cd_SCC and Cd treatments, whereas Cd_CN displayed intermediate patterns between the two. The volcano plot (**Supplementary Figure 1**) analysis illustrated the distribution of DEGs in terms of significance and fold change, and the Cd_SCC VS. Cd comparison had the largest number of significantly upregulated genes. Additional GO and KEGG enrichment analyses were conducted based on the expression of these DEGs.

#### 3.4.3 GO functional enrichment analysis

**Figure 8:**
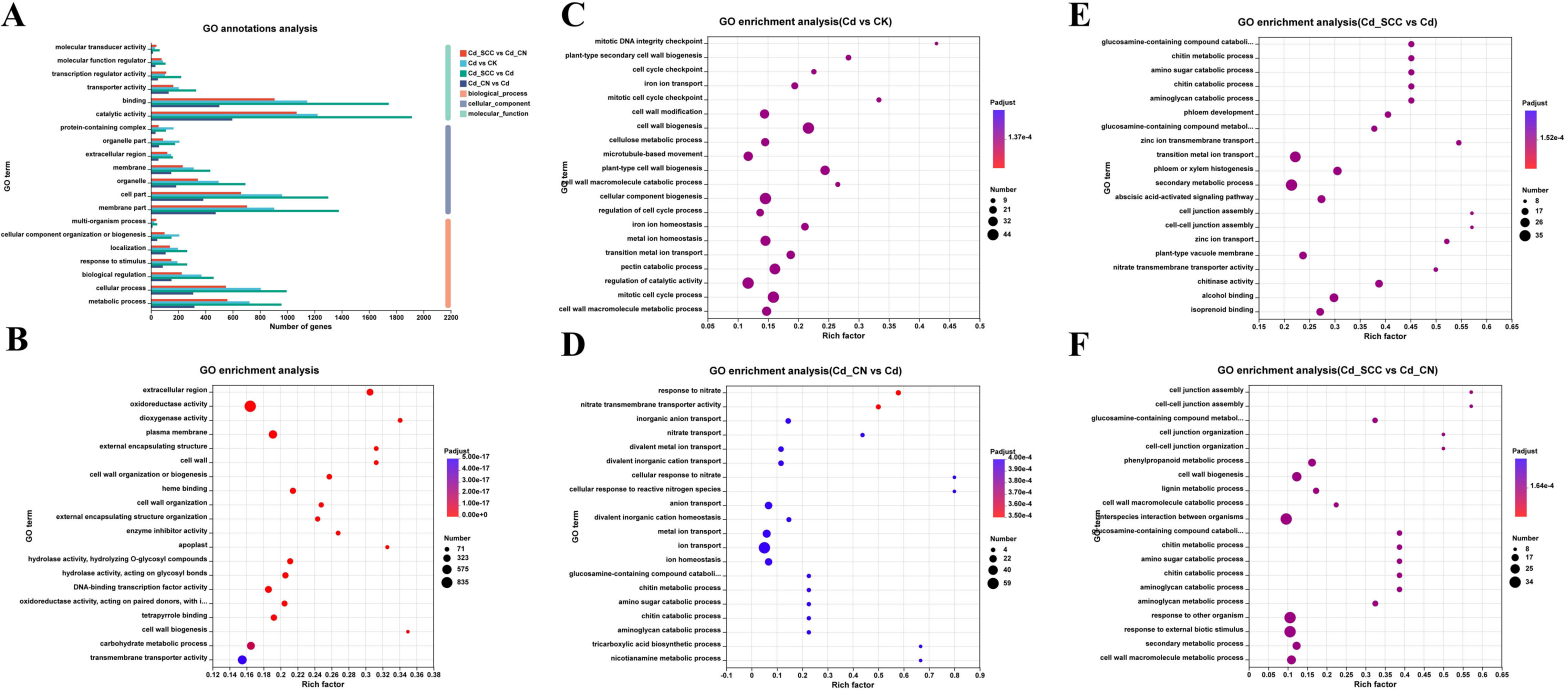
GO functional annotation analysis of differentially expressed genes under different treatments. **(A)** GO classification of DEGs; **(B)** summary comparison of GO enrichment results; **(C)** GO enrichment analysis of Cd VS. CK; **(D)** GO enrichment analysis of Cd_CN VS. Cd; **(E)** GO enrichment analysis of Cd_SCC VS. Cd; **(F)** GO enrichment analysis of Cd_SCC VS. Cd_CN.

The effects of exogenous Ca on the functional distribution of root genes in peanut seedlings under Cd stress were determined by performing a GO functional annotation of the DEGs (**Figure 8A**). The DEGs between the Cd and CK treatments were primarily classified into three major GO categories, including biological process (BP), cellular component (CC), and molecular function (MF). The DEGs in the BP category were mainly involved in metabolic process (GO:0008152), biological regulation (GO:0065007), and cellular process (GO:0009987). In contrast, the DEGs in the CC category were largely associated with cellular component (GO:0044464), membrane component (GO:0044425), organelle (GO:0043226), and membrane (GO:0016020). The DEGs in the MF category were enriched in catalytic activity (GO:0003824), binding (GO:0005488), transporter activity (GO:0005215), and antioxidant activity (GO:0016209).

The annotation results of the two Ca treatments showed that 3,352, 4,369, and 4,479 DEGs were annotated in the BP, CC, and MF categories in the Cd_SCC VS. Cd comparison, respectively. These values were much higher than those in the Cd_CN VS. Cd comparison (1,079, 1,380, and 1,362 DEGs, respectively) (**Supplementary Table 3**).

An additional GO enrichment analysis of the DEGs (**Figures 8B–F**) revealed distinct functional trends. The DEGs between the Cd and CK were primarily enriched in redox processes, stress response, metal ion transport, and cell wall organization (**Figure 8C**). The DEGs between Cd_CN and Cd were mainly involved in nitrate transport, inorganic anion transport, and cellular response to ROS (**Figure 8D**), while the DEGs between Cd_SCC and Cd were enriched in chitin metabolism, amino sugar metabolism, glucosamine metabolism, and cell junction assembly (**Figure 8E**). In contrast, the DEGs between Cd_SCC and Cd_CN were largely enriched in cell wall biosynthesis, secondary metabolism, and stress response pathways (**Figure 8F**). Overall (**Figure 8B**), the GO enrichment results of all the DEGs were primarily concentrated in core biological processes, such as cell wall composition, redox regulation, transmembrane transport, and catalytic activity.

#### 3.4.4 KEGG functional enrichment analysis

**Figure 9:**
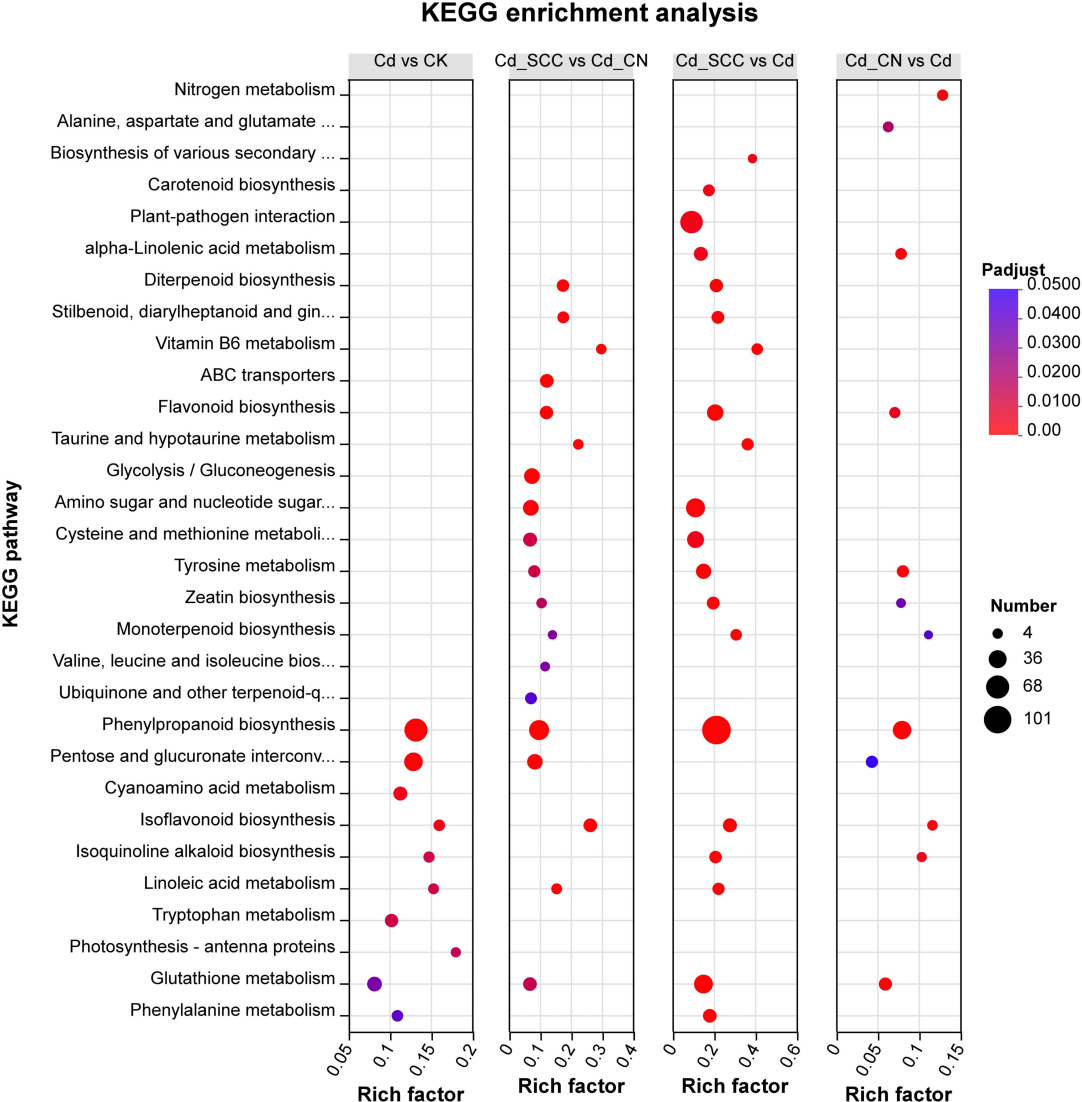
KEGG enrichment analysis of differentially expressed genes in peanut roots under Cd stress and exogenous calcium treatments.

KEGG annotation and enrichment analyses were performed for four pairwise comparisons (Cd vs. CK, Cd_CN vs. Cd, Cd_SCC vs. Cd, and Cd_SCC vs. Cd_CN). The DEGs were broadly involved in multiple primary KEGG pathways, including metabolism, genetic information processing, environmental information processing, and cellular processes (**Supplementary Figure 2**). The top 20 significantly enriched pathways (Padjust < 0.05) are shown in **Figure 9**. In Cd VS. CK (**Supplementary Figure 3A**), the DEGs were mainly enriched in carbon metabolism, amino acid metabolism, secondary metabolite biosynthesis, and energy metabolism. The DEGs in Cd_CN VS. Cd (**Supplementary Figure 3B**) were concentrated in carbon (C) metabolism, amino acid metabolism, and energy metabolism. The enrichment in Cd_SCC VS. Cd (**Supplementary Figure 3C**), was broader and covered not only the metabolic pathways described above but also glutathione metabolism, redox processes, and signal transduction. The DEGs in Cd_SCC VS. Cd_CN (**Supplementary Figure 3D**) were highly enriched in C metabolism, amino acid metabolism, lipid metabolism, cell wall biosynthesis, signal transduction, and environmental adaptation pathways.

**Figure 10:**
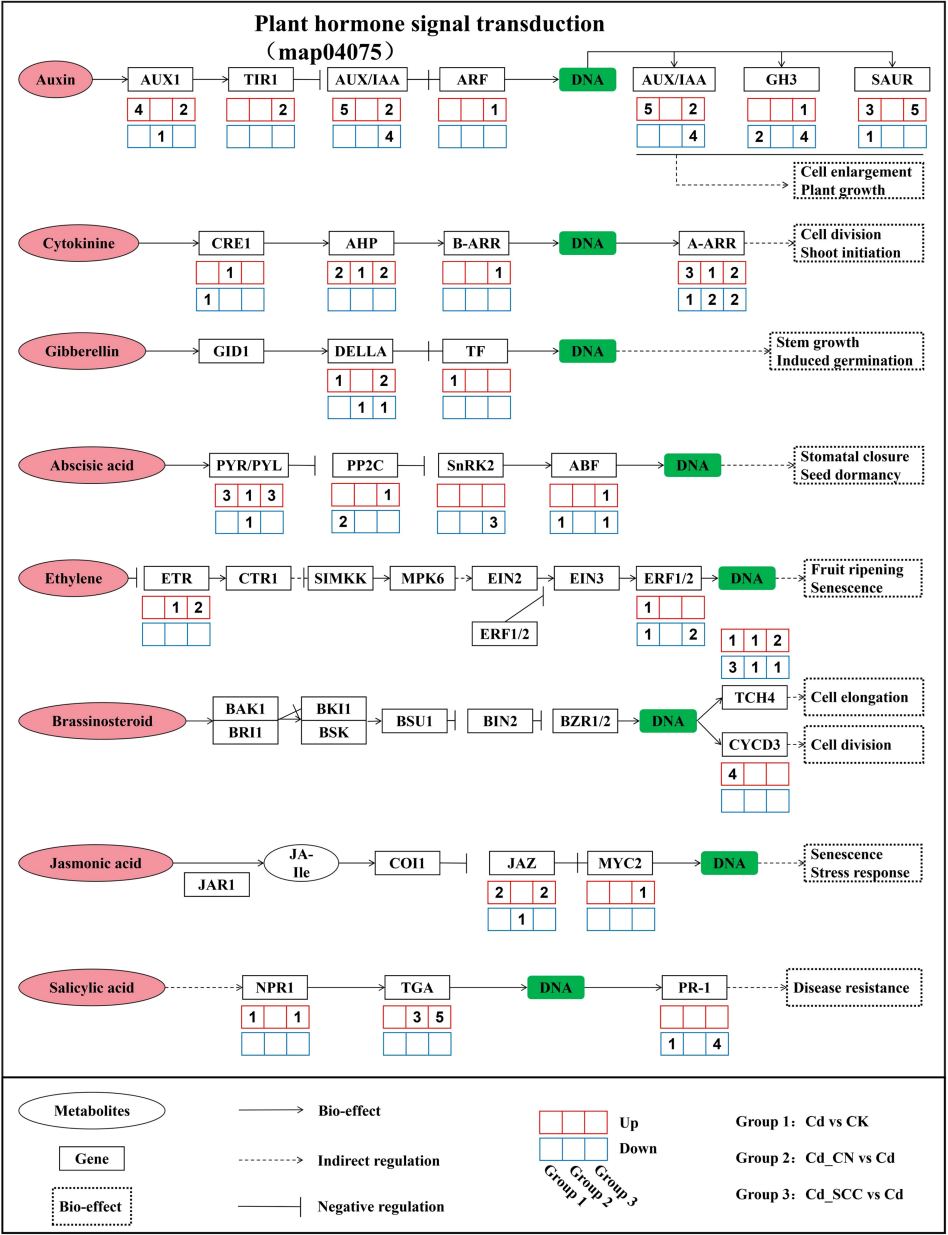
Analysis of plant hormone signal transduction pathways.

Among these pathways, *plant hormone signal transduction* (map04075) was a key stress-related pathway (**Figure 10**). Cd stress markedly inhibited the hormone signalling pathways related to the promotion of growth, such as auxin (IAA), cytokinin (CK), and gibberellin (GA), while also disrupting normal defence-related hormone signalling, including ethylene (ET), jasmonic acid (JA), and salicylic acid (SA). The application of inorganic (Cd_CN vs. Cd) partially restored several hormone pathways, such as AHP and B-ARR in the cytokinin pathway, and PP2C and SnRK2 in the abscisic acid (ABA) pathway. These findings suggest that inorganic Ca helps to mitigate the disruption of hormone signalling induced by Cd.

In contrast, SCC exhibited more pronounced regulatory effects on hormone signal transduction. In the auxin pathway, key genes, such as *AUX/IAA*, *GH3*, and *SAUR*, were greatly upregulated. This promoted cell elongation and division. In addition, *ERF*, *JAZ*, *TGA*, and *PR-1* were strongly activated in the pathways related to defence that are associated with ET, JA, and SA. Therefore, SCC enhanced the resistance of the plant to stress and its ability to defend itself.

Overall, Cd stress not only suppressed the pathways related to metabolism but also disrupted hormone signalling. The application of exogenous Ca alleviated these effects, and SCC caused a stronger response than inorganic Ca as SCC simultaneously promoted the hormone signalling related to growth and activated the hormone pathways related to defence. This combination caused a dual balance between growth and stress tolerance under Cd stress.

#### 3.4.5 Analysis of the transcription factors

**Figure 11:**
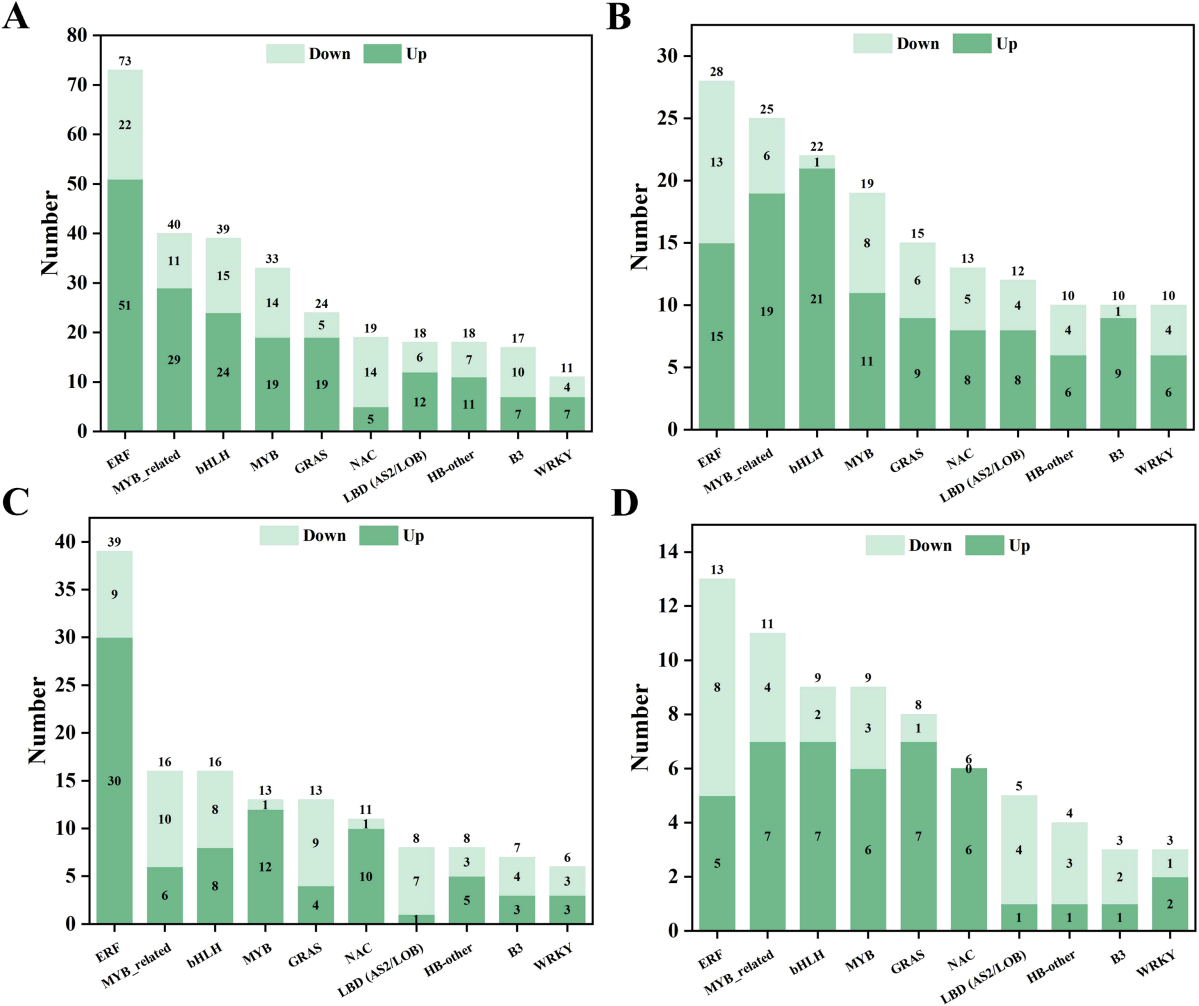
Classification and distribution of differentially expressed transcription factor families under different treatments. **(A)** Statistics of differentially expressed transcription factors in Cd vs. CK; **(B)** Cd_CN vs. Cd; **(C)** Cd_SCC vs. Cd; **(D)** Cd_SCC vs. Cd_CN.

The transcription factor (TF) family statistics were analysed based on the DEGs (**Figure 11**). The results showed that the major TF families included ERF, bHLH, MYB, and WRKY. The ERF family contained the largest number of DEGs (28) in the Cd VS. CK comparison (**Figure 11A**), followed by the MYB-related (25) and bHLH (22) families. In the Cd_CN VS. Cd comparison (**Figure 11B**), the ERF family had 73 members, while the MYB-related, bHLH, and MYB families contained 40, 39, and 33 members, respectively. There was a relatively small total number of differential TFs in the Cd_SCC VS. Cd comparison (**Figure 11C**). There were 13, 11, and 9 members in the ERF, MYB, and bHLH families, respectively. In the Cd_SCC VS. Cd_CN comparison (**Figure 10D**), the ERF family accounted for 39 DEGs, followed by the MYB (16), bHLH (13), and WRKY (13) families.

### 3.5 Integrated analysis

**Figure 12:**
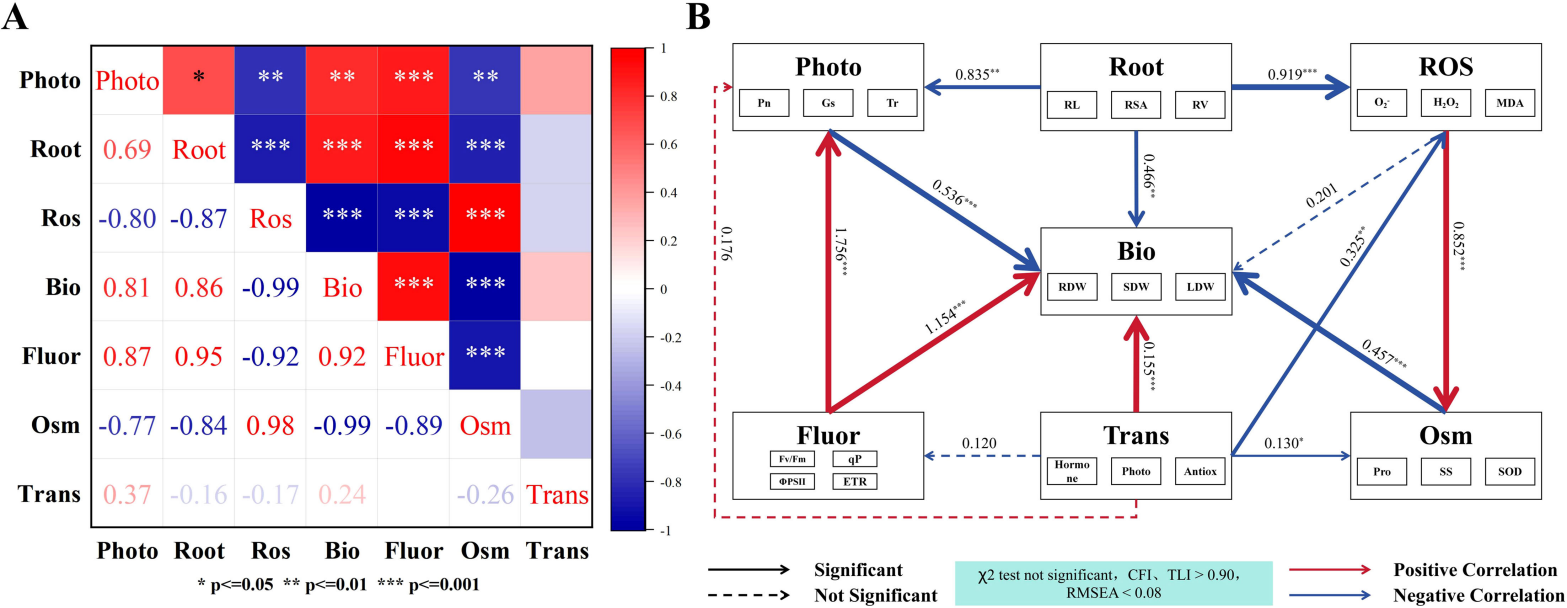
Correlation analysis and structural equation modelling (SEM). **(A)** Correlation heatmap among different parameters (red indicates positive correlation, blue indicates negative correlation); **(B)** structural equation model illustrating the relationships between biomass (Bio: SDW, stem dry weight; LDW, leaf dry weight; RDW, root dry weight) and root structure (Root: RL, total root length; RSA, root surface area; RV, root volume), photosynthesis (Photo: Pn, net photosynthetic rate; Gs, stomatal conductance; Tr, transpiration rate), reactive oxygen species level (ROS: O_2_^-^, superoxide anion; H_2_O_2_, hydrogen peroxide; MDA, malondialdehyde), chlorophyll fluorescence (Fluor: Fv/Fm, maximum quantum efficiency of PSII; ΦPSII, effective quantum yield of PSII; ETR, electron transport rate; qP, photochemical quenching coefficient), osmotic regulation (Osm: SS, soluble sugar; Pro, proline; SP, soluble protein), and transcriptome (Trans: Hormone: Hormone-related genes, Photo: Photosynthesis-related genes, Antiox: Antioxidant-related genes).

A correlation analysis (**Figure 12A**) and structural equation modelling (SEM) (**Figure 12B**) were performed to further reveal the relationships among different physiological indicators and their regulatory effects on the biomass.

The correlation heatmap (**Figure 12A**) showed strong associations among the various parameters. The biomass highly correlated with the root structure (Root, *r* = 0.86***) and chlorophyll fluorescence (Fluor, *r* = 0.92***) in a positive manner, and there was a strong positive correlation with photosynthesis (Photo, *r* = 0.81**). In contrast, it showed highly negative correlations with osmotic regulation (Osm, *r* = −0.99***) and the levels of reactive oxygen species (ROS, *r* = −0.99***). These results indicate that the root development, fluorescence characteristics, and photosynthetic capacity are conducive to the accumulation of biomass, whereas the excessive accumulation of osmolytes and ROS significantly inhibits it. The ROS is strongly and positively correlated with Osm (*r* = 0.98***), which suggests that the accumulation of ROS is accompanied by an increase in the osmotic regulatory substances. Root has a highly positively correlation with Fluor (*r* = 0.95***). This revealed that there is a close relationship between the development of roots and amount of fluorescence.

The constructed SEM model (**Figure 12B**) showed a good fit (non-significant χ² test, CFI > 0.90, TLI > 0.90, RMSEA < 0.08), which pointed out the direct and indirect effects of each factor on the formation of biomass. Fluor had the strongest direct positive effect on Bio (β = +1.154***), while Photo (β = −0.536***), Root (β = −0.466**), and Osm (β = −0.457***) had significant negative effects. Trans (β = +0.155***) had a smaller but significant positive effect on Bio, whereas ROS (β = −0.201, ns) had no significant direct effect. Overall, Fluor was identified as the core factor that promoted the accumulation of biomass, while the excessive accumulation of ROS and osmotic compounds represented key inhibitory factors that limited the formation of biomass.

## 4 Discussion

### 4.1 Exogenous SCC improves the growth and root morphology of peanut seedlings under Cd stress

When plants are exposed to Cd stress, their growth is typically stunted, and the accumulation of biomass is reduced (Zhang et al., 2023). In this study, treatment with Cd significantly decreased the plant height and content of dry matter in peanut seedlings, which is a typical manifestation of Cd toxicity (Haider et al., 2021). Previous studies have shown that the application of Ca can mitigate such growth inhibition. For example, the use of Ca has been reported to increase the peanut biomass by 61.64% at the podding stage and root biomass by 22.95% at maturity under Cd stress (Zhang et al., 2022). In this study, the addition of 5 mmol/L SCC partially restored the plant height and dry weights of the roots, stems, and leaves. This improvement may be attributed to the participation of SCC in energy metabolism, its interactions with beneficial elements, and its role in alleviating the mitotic abnormalities induced by Cd (Wenling et al., 2020), thereby reducing damage and enhancing plant growth under Cd stress.

In addition to inhibiting the aboveground growth, Cd stress also adversely affects the root morphology (Chen S. et al., 2023). In this study, treatment with Cd suppressed the total root length, surface area, and volume of the peanut seedlings. These responses were probably owing to the inhibition of cell division in the root tips and damage to the root cell structures (Seth et al., 2008; Yuan et al., 2005). Although Cd suppressed the elongation of roots, it promoted their radial thickening. Notably, SCC was more effective than Ca nitrate in alleviating the damage to roots induced by Cd and assisting the growth of roots. This may be because SCC is more readily absorbed and utilized by plants (Li et al., 2022a), thereby strengthening the cell wall structure and stability, limiting the penetration of Cd, and reducing injury to the roots. Moreover, sorbitol in the chelated complex can enter the cells via specific transport proteins and participate in metabolic processes, further promoting the development of roots (Li et al., 2012).

### 4.2 Exogenous calcium improves photosynthesis in peanut seedlings under Cd stress

The reduction in the content of chlorophyll and inhibition of photosynthesis are among the most prominent symptoms of Cd toxicity in plants (An et al., 2019). In this study, the Cd treatment significantly decreased the contents of chlorophyll a, carotenoids, total chlorophyll (Chl a + b), and the Chl a/b ratio. Cd stress damaged the chloroplast structure, accelerated the degradation of chlorophyll, and inhibited the activity of protochlorophyllide reductase, which, in turn, affected carbon fixation and the biosynthesis of γ-aminolevulinic acid. Thus, this ultimately reduced the biosynthesis of chlorophyll (Siddiqui et al., 2022). In contrast, the application of SCC partially restored the levels of chlorophyll and carotenoids, which helped to maintain the ability to conduct photosynthesis and grow under Cd stress. Similar results were reported by Andosch et al (Andosch et al., 2012), who found that Ca alleviated the ultrastructural damage to the chloroplasts and improved photosynthetic performance in algae stressed with Cd. Cd stress also reduced the net Pn, Tr, Gs, and chlorophyll fluorescence intensity, while increasing the Ci. This indicates that the inhibition of photosynthesis by Cd is primarily owing to non-stomatal factors – specifically, a reduction in the photosynthetic activity of mesophyll cells. After exogenous Ca was applied, Pn, Tr, Gs, Fv/Fm, ΦPSII, qP, and ETR were all greatly increased, whereas Ci, F_0_, and qN were markedly decreased. This ensured that the photosynthetic process continued to function properly. These results are consistent with previous findings in peanut and other crops (Gao et al., 2011; Li et al., 2015), which demonstrated that supplementation with Ca effectively improves the photosynthetic efficiency under Cd stress. The Ca in chloroplasts can consume NADPH and glutathione, thereby modifying the redox state within the organelle (Liu, 2015). Moreover, Ca competes with Cd for the adsorption sites on root surfaces, which reduces the uptake of Cd and mitigates its inhibitory effects. Together, these mechanisms explain why exogenous Ca, particularly in chelated form, promotes photosynthesis and stabilizes the function of chloroplasts in peanut leaves stressed by Cd.

### 4.3 Exogenous calcium alleviates the oxidative stress induced by Cd

The excessive accumulation of ROS, such as O_2_^-^ and H_2_O_2_, is one of the main causes of the oxidative damage in plants induced by Cd. This leads to increased membrane permeability, a higher content of MDA, reduction in root activity, and severe cellular injury (Wang et al., 2015). In this study, tissue staining and a quantitative analysis showed that exposure to Cd caused varying degrees of membrane damage in the peanut roots, stems, and leaves. The addition of exogenous Ca, particularly SCC, significantly reduced the contents of O_2_^-^, H_2_O_2_, and MDA, as well as the relative conductivity. These results indicate that treatment with exogenous Ca can effectively scavenge excess ROS in plants stressed by Cd, reduce the peroxidation of lipids and electrolyte leakage, and ultimately mitigate the cellular damage induced by Cd (Li et al., 2023).

Plants under heavy metal stress often regulate osmotic adjustment compounds to resist toxicity. In this study, Cd stress increased the content of soluble protein in peanut tissues, which may be owing to enhanced protease activity and the biosynthesis of Cd-binding peptides, such as phytochelatins (Martínez et al., 2006), metallothioneins (Talebi et al., 2019), and other metal-chelating proteins (Bartolf et al., 1980), that sequester Cd ions and reduce toxicity. However, the addition of SCC lowered the levels of soluble proteins under Cd stress, which suggests that it helps to maintain the stability of protein structure and prevent the degradation of excessive proteins. Soluble sugars and proline are also key osmolytes that balance the osmotic potential under stress conditions (Kreuzwieser and Rennenberg, 2014). In this study, Cd treatment led to significant increases in the contents of soluble sugars and proline in peanut tissues, which helps to maintain the osmotic balance under Cd stress. After supplementation with Ca, these contents were significantly reduced (Torun et al., 2024). This indicated that SCC can alleviate the programmed cell death induced by Cd and regulate the osmotic pressure (Yan, 2020), thereby enhancing the stress tolerance of peanut seedlings.

Cd also altered the activity of antioxidant enzymes in different ways. The activity of SOD decreased, whereas that of POD and CAT increased. This implies that Cd stress affects the transcriptional regulation of the genes for antioxidant enzymes and disrupts redox homeostasis (Rodríguez-Serrano et al., 2009). In this study, SCC effectively boosted the activity of SOD and reduced the accumulation of ROS. This suggests that it activates the first line of defence in the ROS-scavenging system (Sanchez-Casas and Klessig, 1994), thereby reducing the oxidative damage caused by superoxide radicals. Furthermore, as the levels of H_2_O_2_ decreased significantly after SCC treatment, the activities of POD and CAT – the enzymes primarily responsible for the decomposition of H_2_O_2_ – were also reduced. This reflected that the redox balance was restored within the cells.

### 4.4 Exogenous calcium regulates the plant hormone signal transduction pathways in peanut roots under Cd stress

Plant hormones are small signalling molecules that play crucial regulatory roles in mediating plant responses to abiotic stress (Waadt et al., 2022). In this study, exogenous treatment with Ca induced significant transcriptional changes in the genes involved in multiple hormone metabolic pathways under Cd stress, including IAA, CK, GA, ABA, ET, BR, JA, and SA pathways. SCC activated the expression of auxin influx carriers, auxin-responsive proteins, GH3 family proteins, and SAUR family proteins, which help to alleviate the oxidative stress induced by Cd and promote the elongation of roots and cell expansion in peanut seedlings. Cytokinin generally exhibits a negative regulatory effect on stress signalling, which contributes to enhanced stress tolerance in plants (Yan et al., 2021). In this study, exogenous SCC upregulated the AHP and type-B ARR TFs, thereby downregulating the endogenous cytokinin signalling and improving the Cd tolerance in peanut roots. Gibberellins can improve the tolerance to heavy metals by strengthening the antioxidant defences, modulating the compartmentalization of metals, and regulating the cross-talk with other hormone signalling pathways via DELLA proteins (Zhang et al., 2020). In this study, SCC induced the expression of DELLA proteins in the GA signalling pathway, which subsequently released the TFs that contribute to Cd tolerance and root development (Sun, 2020). Under abiotic stress, elevated levels of ABA can strengthen the tolerance of plants to heavy metals. SCC treatment significantly upregulated the ABA receptor proteins (PYR/PYL), which reinforced resistance to Cd. Moreover, the increased endogenous ABA content induced by SCC may regulate the biosynthesis of glutathione (GSH) (Li et al., 2014) and activity of phytochelatin synthase (PCS) (Sharma and Kumar, 2002), which contributes to the detoxification of Cd and maintenance of redox homeostasis. Brassinosteroids (BRs) also play key roles in plant defence against abiotic stress (Chaudhuri et al., 2022). In this study, SCC upregulated the expression of *TCH4* (*xyloglucan endotransglucosylase/hydrolase*) in the BR pathway, which promoted loosening of the cell wall and elongation of the root cells under Cd stress. Jasmonic acid (JA) is another essential hormone involved in abiotic stress responses (Howe et al., 2018). In *Arabidopsis thaliana*, mutation of the JA biosynthetic gene *AtAOS* causes the overexpression of *AtIRT1*, *AtHMA2*, and *AtHMA4*, which reduces the uptake of Cd and toxicity (Lei et al., 2020). Similarly, SCC enhanced the expression of *JAZ (Jasmonate ZIM-domain)* proteins and promoted the activation of the TF *MYC2*, thereby improving the tolerance to Cd in peanut roots. Furthermore, an analysis of the TFs revealed that the ERF, MYB, and bHLH families were significantly upregulated in the Cd_SCC treatment. These TFs are tightly coupled with hormone signalling networks and regulate the key downstream genes involved in indole-3-acetic acid (IAA), JA, and SA pathways. Therefore, SCC not only modulates hormone signalling at the metabolic level but also strengthens the transcriptional regulation networks, thus, jointly enhancing the Cd tolerance in peanut roots.

Collectively, the transcriptomic results indicate that SCC enhances Cd tolerance in peanut roots by coordinately modulating multiple plant hormone signalling pathways. Among these pathways, regulatory modules related to IAA, ABA, and JA appear to play key roles in integrating root growth regulation with heavy metal detoxification. Based on the findings of this study, future research could focus on the key regulatory nodes within these hormone signalling pathways and their roles in controlling root architecture, antioxidant defence, and Cd detoxification processes, which could further elucidate the molecular basis underlying chelated calcium–mediated alleviation of Cd stress in plants.

### 4.5 Integrated analysis

Under Cd stress, both promoting and inhibiting factors jointly influence plant growth. Chlorophyll fluorescence strongly promotes the accumulation of biomass, whereas excessive ROS and osmotic regulators act as key limiting factors (Naciri et al., 2021). Centred on biomass, the SEM clearly revealed two major pathways – a growth-promoting axis and a growth-suppressing axis. The primary driver on the growth-promoting axis was chlorophyll fluorescence, which not only directly contributed to biomass formation but also enhanced the photosynthetic efficiency by stabilizing PSII and electron transport. This led to more effective assimilation of C (Murchie and Lawson, 2013). In contrast, the root system did not increase the biomass through a direct route. Instead, it played an upstream regulatory role under stress. The negative path from “Root” to “ROS” indicated that healthier root systems reduced the accumulation of ROS and relieved pressure on the photosynthetic apparatus (Tarkowski et al., 2023). Because the accumulation of ROS further increased osmotic and Osm, which, in turn, suppressed biomass production (Shahid et al., 2020), the root’s essential contribution was realized through the indirect path of “Root → lower ROS → lower Osm → promote growth (Liu et al., 2022).”

At the transcriptional level, regulation primarily occurred through the integration of hormone signalling, upregulation of genes related to photosynthesis, and activation of the antioxidant enzymes, such as CAT. Together, they formed a molecular feedback loop of “stress relief – photosynthetic stabilization – growth promotion,” which indirectly enhanced the accumulation of biomass. Notably, the SEM revealed the presence of “mediated inhibitory effects.” The introduction of fluorescence and stress-related variables into the model caused the direct paths from photosynthesis and root traits to biomass to become negative – not because they truly suppressed growth – but because their positive effects were absorbed by strong mediators, such as fluorescence, ROS, and Osm. Similar mediating effects (S and T, 2025) have been demonstrated in previous studies, where the net path coefficients may turn negative once the indirect effects dominate. Furthermore, the inhibitory influence of ROS on biomass was both direct and indirect and primarily expressed through the “ROS → Osm → Bio” pathway. This reflects that energy-consuming defensive metabolism diverts C resources away from the accumulation of biomass (Karasov et al., 2017). Therefore, the most effective strategy to promote growth under Cd stress is to stabilize the photochemical system via enhanced fluorescence, optimize root architecture which suppresses the generation of ROS at the source, and reduces the osmotic and antioxidant metabolic burdens at the molecular level – achieving a synergistic “Fluor↑–Photo stable–ROS↓–Osm↓” pattern that ultimately drives biomass increase.

## 5 Conclusions

Under the conditions of this study, Cd stress significantly inhibited the growth, photosynthesis, and antioxidant system of peanut seedlings. The application of exogenous calcium effectively alleviated these adverse effects. SCC was the most effective at mitigating these negative effects. Compared with inorganic calcium, SCC more effectively promoted the development of growth traits, such as plant height and stem diameter, optimized root structure, enhanced the accumulation of dry matter, and raised the contents of photosynthetic pigments and characteristics of chlorophyll fluorescence. In addition, SCC reduced the excessive accumulation of ROS and osmotic regulators and lowered the peroxidation of membrane lipids by regulating the activities of antioxidant enzymes. At the transcriptional level, SCC induced a larger number and broader range of differentially expressed genes, which were significantly enriched in pathways related to cell wall biosynthesis, secondary metabolism, and plant hormone signal transduction. These changes collectively promoted both growth and defence, forming a dual “growth–stress resistance” regulatory mechanism. Integrated correlation and SEM analyses further revealed that chlorophyll fluorescence was the key driver of biomass accumulation, whereas ROS and osmotic regulation were the main limiting factors. Overall, SCC was much more effective at mitigating Cd toxicity, enhancing stress tolerance, and promoting the growth and development of peanut seedlings compared with inorganic calcium.

## Data Availability

The raw RNA-seq data generated in this study have been deposited in the NCBI Sequence Read Archive (SRA) repository under BioProject accession number PRJNA1435608.
