# Supplementary material for: Exogenous sorbitol-chelated calcium mitigates toxicity of cadmium in peanut seedlings through physiological, biochemical, and transcriptomic regulation
Source: Front Plant Sci. 2026 Mar 23;17:1741995. doi: 10.3389/fpls.2026.1741995 (PMC13050962; doi:10.3389/fpls.2026.1741995)
Supplement: Supplementary file 1 [file DataSheet1.docx]

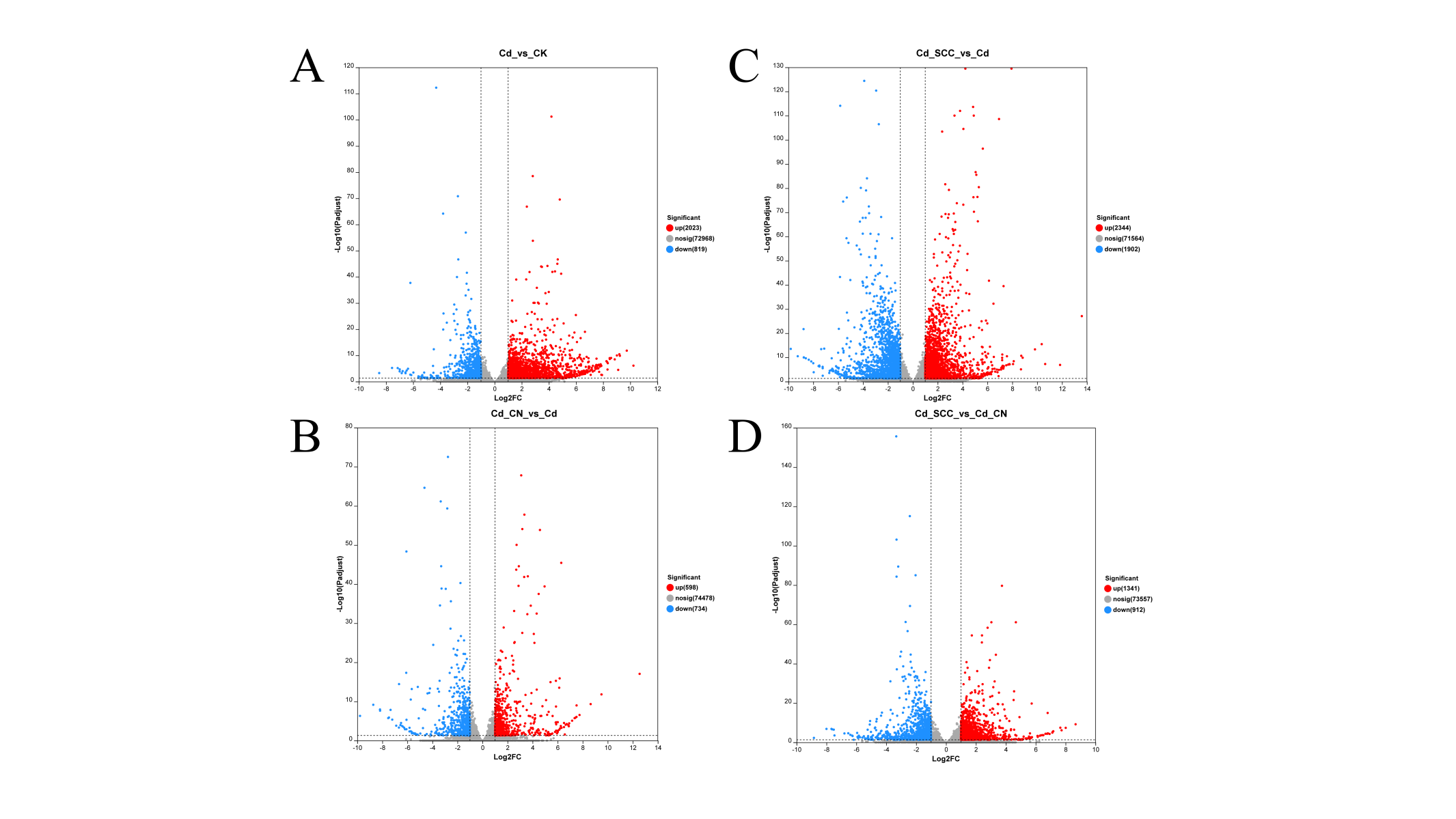


**Fig S1 Volcano plot of differentially expressed genes in peanut seedling roots under different treatments**

Note: (A) Cd vs CK; (B) Cd_CN vs Cd; (C) Cd_SCC vs Cd; (D) Cd_SCC vs Cd_CN. The horizontal axis represents log₂(Fold Change), and the vertical axis represents –log₁₀(FDR). Red dots indicate significantly upregulated genes, blue dots indicate significantly downregulated genes, and gray dots indicate genes with no significant difference.

**
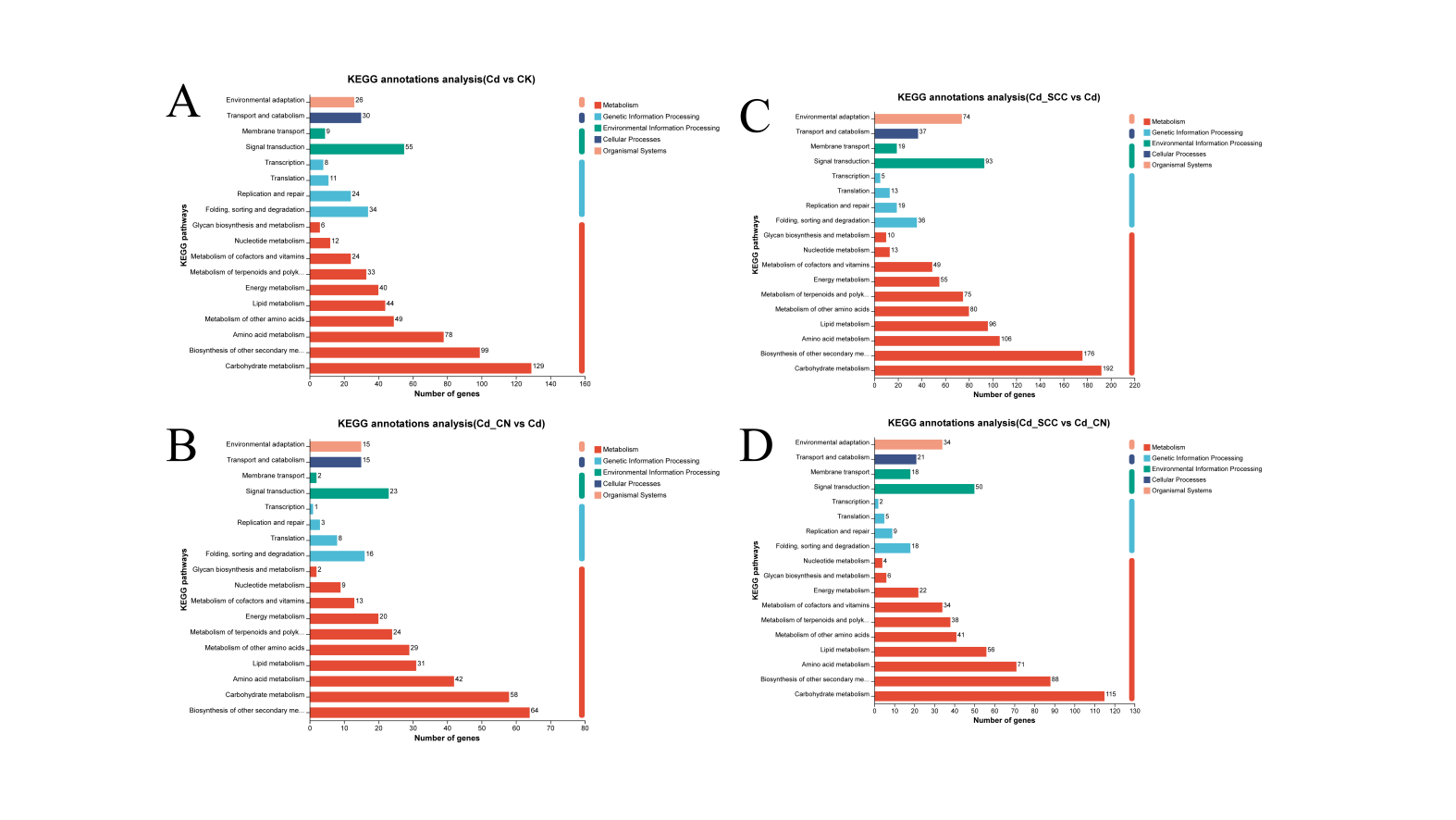
**

**Fig S2 KEGG annotation classification of differentially expressed genes in peanut roots under Cd stress and exogenous calcium treatments**

Note: (A) Cd vs CK; (B) Cd_CN vs Cd; (C) Cd_SCC vs Cd; (D) Cd_SCC vs Cd_CN.

**
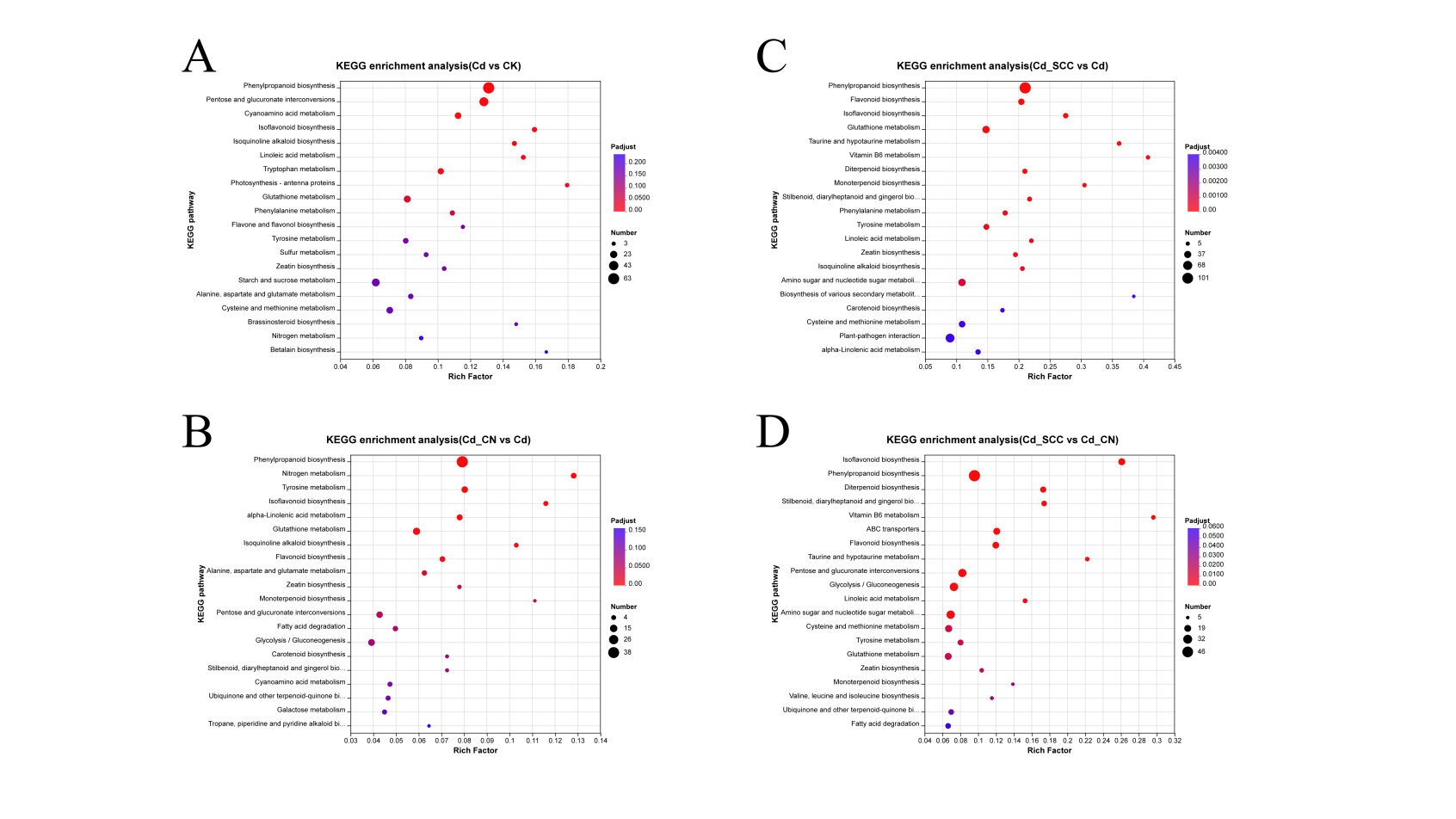
**

**Fig S3 KEGG enrichment analysis of differentially expressed genes in peanut roots under Cd stress and exogenous calcium treatments**

Note: (A) Cd vs CK; (B) Cd_CN vs Cd; (C) Cd_SCC vs Cd; (D) Cd_SCC vs Cd_CN. The bubble size represents the number of differentially expressed genes, and the color indicates the enrichment significance level (Padjust value).
