# Supplementary material for: Exogenous sorbitol-chelated calcium mitigates toxicity of cadmium in peanut seedlings through physiological, biochemical, and transcriptomic regulation
Source: Front Plant Sci. 2026 Mar 23;17:1741995. doi: 10.3389/fpls.2026.1741995 (PMC13050962; doi:10.3389/fpls.2026.1741995)
Supplement: Supplementary file 2 [file Table1.docx]

| **Sample** | **Plant height (cm)** | **Stem diameter (mm)** | **Root dry weight (g)** | **Stem dry weight (g)** | **Leaf dry weight (g)** | **RL (cm)** | **TRL (cm)** | **RSA (cm2)** | **RAD (mm)** | **RV (cm3)** |
| --- | --- | --- | --- | --- | --- | --- | --- | --- | --- | --- |
| Cd | 9.95±0.44c | 2.15±0.17d | 0.113±0.006e | 0.268±0.017d | 0.149±0.018d | 9.28±0.48c | 117.93±0.36e | 32.54±0.24c | 0.88±0.01bc | 0.71±0.01d |
| Cd_SCC1 | 12.53±0.25b | 2.93±0.25a | 0.186±0.011d | 0.340±0.013bc | 0.221±0.011b | 12.15±1.39b | 197.95±4.92d | 56.79±1.37b | 0.92±0.01b | 1.18±0.11c |
| Cd_SCC2.5 | 13.93±1.09a | 2.70±0.08b | 0.247±0.002c | 0.362±0.035bc | 0.247±0.003a | 12.50±0.94b | 237.61±17.48b | 59.25±5.67ab | 0.85±0.04bc | 1.37±0.14ab |
| Cd_SCC5 | 14.65±0.60a | 2.70+0.08b | 0.314±0.019a | 0.418±0.013a | 0.265±0.025a | 15.90±0.57a | 258.77±17.99a | 66.46±3.64a | 0.82±0.05c | 1.44±0.08a |
| Cd_SCC10 | 9.75±0.41c | 2.43±0.05c | 0.263±0.010bc | 0.369±0.025b | 0.185±0.007c | 15.00±1.44a | 216.91±1.18c | 53.31±6.90b | 0.75±0.03d | 1.24±0.11bc |
| Cd_SCC20 | 7.48±0.25d | 1.40±0.08e | 0.269±0.001b | 0.329±0.013c | 0.148±0.012d | 9.33±0.60c | 136.03±9.52e | 39.30±3.74c | 1.06±0.09a | 0.65±0.02d |

**Table S1 Effects of 0–20 mM sorbitol-chelated calcium (SCC) on the growth parameters of peanut seedlings under Cd stress (2.5 mg·kg⁻¹).**

Note:Results are expressed as mean ± standard deviation (n = 3). Different lowercase letters indicate significant differences at P < 0.05 based on Duncan’s test. RL, TRL, RSA, RAD, and RV represent root length, total root length, root surface area, average root diameter, and root volume, respectively.

| **Sample** | **Raw**  **reads** | **Raw**  **bases** | **Clean**  **reads** | **Clean**  **bases** | **Error rate**  **(%)** | **Q20**  **(%)** | **Q30**  **(%)** | **GC content**  **(%)** | **Total**  **reads** | **Total**  **mapped** | **Multiple**  **mapped** | **Uniquely**  **mapped** |
| --- | --- | --- | --- | --- | --- | --- | --- | --- | --- | --- | --- | --- |
| CK1 | 56085160 | 8468859160 | 54061508 | 7733156882 | 0.0269 | 97.4 | 92.53 | 45.6 | 54061508 | 50228562  (92.91%) | 7374992  (13.64%) | 42853570  (79.27%) |
| CK2 | 49231164 | 7433905764 | 47709728 | 6833906132 | 0.0269 | 97.39 | 92.48 | 45.52 | 47709728 | 44652743  (93.59%) | 6270761  (13.14%) | 38381982  (80.45%) |
| CK3 | 55143476 | 8326664876 | 53122592 | 7665580110 | 0.0272 | 97.27 | 92.24 | 45.93 | 53122592 | 49575681  (93.32%) | 6593997  (12.41%) | 42981684  (80.91%) |
| Cd1 | 61844442 | 9338510742 | 58833664 | 8514306843 | 0.0268 | 97.41 | 92.61 | 45.48 | 58833664 | 55023947  (93.52%) | 7563994  (12.86%) | 47459953  (80.67%) |
| Cd2 | 53946788 | 8145964988 | 51380100 | 7510782575 | 0.0272 | 97.24 | 92.21 | 45.6 | 51380100 | 47630473  (92.7%) | 5948933  (11.58%) | 41681540  (81.12%) |
| Cd3 | 56054454 | 8464222554 | 53236250 | 7726904485 | 0.0279 | 96.96 | 91.7 | 45.35 | 53236250 | 50026824  (93.97%) | 6850314  (12.87%) | 43176510  (81.1%) |
| Cd_CN1 | 46578258 | 7033316958 | 45217300 | 6541323941 | 0.0264 | 97.58 | 92.88 | 46.45 | 45217300 | 42603770  (94.22%) | 5675933  (12.55%) | 36927837  (81.67%) |
| Cd_CN2 | 49722970 | 7508168470 | 46574242 | 6810149148 | 0.0268 | 97.39 | 92.56 | 45.32 | 46574242 | 43933927  (94.33%) | 5405621  (11.61%) | 38528306  (82.72%) |
| Cd_CN3 | 51805662 | 7822654962 | 50337890 | 7332190290 | 0.0268 | 97.42 | 92.58 | 45.87 | 50337890 | 47768851  (94.9%) | 6230271  (12.38%) | 41538580  (82.52%) |
| Cd_SCC1 | 50416056 | 7612824456 | 48709974 | 7028721719 | 0.0269 | 97.39 | 92.52 | 45.89 | 48709974 | 46267417  (94.99%) | 6052365  (12.43%) | 40215052  (82.56%) |
| Cd_SCC2 | 51445660 | 7768294660 | 50122124 | 7270356174 | 0.0269 | 97.39 | 92.5 | 45.45 | 50122124 | 47216884  (94.2%) | 6270959  (12.51%) | 40945925  (81.69%) |
| Cd_SCC3 | 47374316 | 7153521716 | 45180438 | 6612851461 | 0.0269 | 97.33 | 92.44 | 45.48 | 45180438 | 42504340  (94.08%) | 5020361  (11.11%) | 37483979  (82.97%) |

**Table S2 RNA-seq sequencing and alignment quality statistics.**

Note: The table lists the Raw reads/Raw bases, Clean reads/Clean bases and their ratios, error rate, Q20/Q30, GC content, and alignment results (Total reads = Clean reads, Total mapped, Uniquely mapped, and Multiple mapped; percentages in parentheses indicate proportions of Total reads), which are used to evaluate the sequencing data quality and alignment reliability.

| **GOID** | **Description** | **TermType** | **Cd_SCC_vs_Cd_CN_Gnum** | **Cd_vs_CK_Gnum** | **Cd_CN_vs_Cd_Gnum** | **Cd_SCC_vs_Cd_Gnum** |
| --- | --- | --- | --- | --- | --- | --- |
| GO:0001906 | cell killing | biological_process | 3 | 0 | 2 | 3 |
| GO:0002376 | immune system process | biological_process | 5 | 2 | 1 | 11 |
| GO:0015976 | carbon utilization | biological_process | 0 | 1 | 0 | 1 |
| GO:0065007 | biological regulation | biological_process | 226 | 370 | 151 | 460 |
| GO:0008152 | metabolic process | biological_process | 561 | 723 | 319 | 958 |
| GO:0051704 | multi-organism process | biological_process | 40 | 25 | 12 | 46 |
| GO:0040011 | locomotion | biological_process | 0 | 0 | 1 | 2 |
| GO:0022414 | reproductive process | biological_process | 29 | 55 | 13 | 64 |
| GO:0000003 | reproduction | biological_process | 6 | 8 | 1 | 4 |
| GO:0008283 | cell population proliferation | biological_process | 2 | 2 | 0 | 4 |
| GO:0071840 | cellular component organization or biogenesis | biological_process | 100 | 208 | 46 | 151 |
| GO:0009987 | cellular process | biological_process | 550 | 806 | 310 | 996 |
| GO:0032502 | developmental process | biological_process | 37 | 72 | 13 | 82 |
| GO:0032501 | multicellular organismal process | biological_process | 14 | 21 | 6 | 23 |
| GO:0040007 | growth | biological_process | 3 | 1 | 0 | 5 |
| GO:0048511 | rhythmic process | biological_process | 3 | 0 | 1 | 2 |
| GO:0051179 | localization | biological_process | 139 | 198 | 108 | 266 |
| GO:0022610 | biological adhesion | biological_process | 0 | 2 | 1 | 2 |
| GO:0098754 | detoxification | biological_process | 9 | 6 | 6 | 6 |
| GO:0023052 | signaling | biological_process | 0 | 1 | 1 | 0 |
| GO:0019740 | nitrogen utilization | biological_process | 0 | 1 | 0 | 0 |
| GO:0050896 | response to stimulus | biological_process | 150 | 194 | 87 | 266 |
| GO:0031974 | membrane-enclosed lumen | cellular_component | 2 | 6 | 4 | 9 |
| GO:0032991 | protein-containing complex | cellular_component | 56 | 166 | 34 | 110 |
| GO:0005623 | cell | cellular_component | 17 | 38 | 18 | 37 |
| GO:0044425 | membrane part | cellular_component | 705 | 904 | 475 | 1379 |
| GO:0044421 | extracellular region part | cellular_component | 20 | 11 | 5 | 16 |
| GO:0044422 | organelle part | cellular_component | 88 | 209 | 59 | 177 |
| GO:0043226 | organelle | cellular_component | 344 | 496 | 185 | 693 |
| GO:0016020 | membrane | cellular_component | 233 | 314 | 149 | 435 |
| GO:0030054 | cell junction | cellular_component | 13 | 31 | 4 | 29 |
| GO:0005576 | extracellular region | cellular_component | 120 | 149 | 55 | 161 |
| GO:0009295 | nucleoid | cellular_component | 0 | 1 | 0 | 0 |
| GO:0044217 | other organism part | cellular_component | 3 | 37 | 7 | 14 |
| GO:0044464 | cell part | cellular_component | 661 | 962 | 384 | 1301 |
| GO:0099080 | supramolecular complex | cellular_component | 6 | 42 | 1 | 8 |
| GO:0045182 | translation regulator activity | molecular_function | 5 | 11 | 5 | 14 |
| GO:0140110 | transcription regulator activity | molecular_function | 112 | 103 | 51 | 222 |
| GO:0005198 | structural molecule activity | molecular_function | 11 | 14 | 4 | 14 |
| GO:0044183 | protein folding chaperone | molecular_function | 2 | 3 | 2 | 2 |
| GO:0140104 | molecular carrier activity | molecular_function | 0 | 2 | 0 | 0 |
| GO:0016209 | antioxidant activity | molecular_function | 29 | 44 | 14 | 46 |
| GO:0005215 | transporter activity | molecular_function | 164 | 204 | 130 | 331 |
| GO:0098772 | molecular function regulator | molecular_function | 78 | 88 | 33 | 107 |
| GO:0140299 | small molecule sensor activity | molecular_function | 2 | 2 | 4 | 6 |
| GO:0005488 | binding | molecular_function | 907 | 1146 | 502 | 1745 |
| GO:0045735 | nutrient reservoir activity | molecular_function | 10 | 4 | 5 | 11 |
| GO:0031386 | protein tag | molecular_function | 0 | 0 | 0 | 2 |
| GO:0060089 | molecular transducer activity | molecular_function | 40 | 29 | 15 | 64 |
| GO:0003824 | catalytic activity | molecular_function | 1069 | 1223 | 597 | 1915 |

**Table S3 GO functional annotation of differentially expressed genes (DEGs) in peanut seedling roots.**
